# Structural dynamics at surfaces by ultrafast reflection high-energy electron diffraction

**DOI:** 10.1063/4.0000234

**Published:** 2024-03-12

**Authors:** Michael Horn-von Hoegen

**Affiliations:** Department of Physics and Center for Nanointegration CENIDE, University of Duisburg-Essen, Lotharstrasse. 1, 47057 Duisburg, Germany

## Abstract

Many fundamental processes of structural changes at surfaces occur on a pico- or femtosecond timescale. In order to study such ultrafast processes, we have combined modern surface science techniques with fs-laser pulses in a pump–probe scheme. Grazing incidence of the electrons ensures surface sensitivity in ultrafast reflection high-energy electron diffraction (URHEED). Utilizing the Debye–Waller effect, we studied the nanoscale heat transport from an ultrathin film through a hetero-interface or the damping of vibrational excitations in monolayer adsorbate systems on the lower ps-timescale. By means of spot profile analysis, the different cooling rates of epitaxial Ge nanostructures of different size and strain state were determined. The excitation and relaxation dynamics of a driven phase transition far away from thermal equilibrium is demonstrated using the In-induced (8 × 2) reconstruction on Si(111). This Peierls-distorted surface charge density wave system exhibits a discontinuous phase transition of first order at 130 K from a (8 × 2) insulating ground state to (4 × 1) metallic excited state. Upon excitation by a fs-laser pulse, this structural phase transition is non-thermally driven in only 700 fs into the excited state. A small barrier of 40 meV hinders the immediate recovery of the ground state, and the system is found in a metastable supercooled state for up to few nanoseconds.

## I. INTRODUCTION

**FIG. 1.**
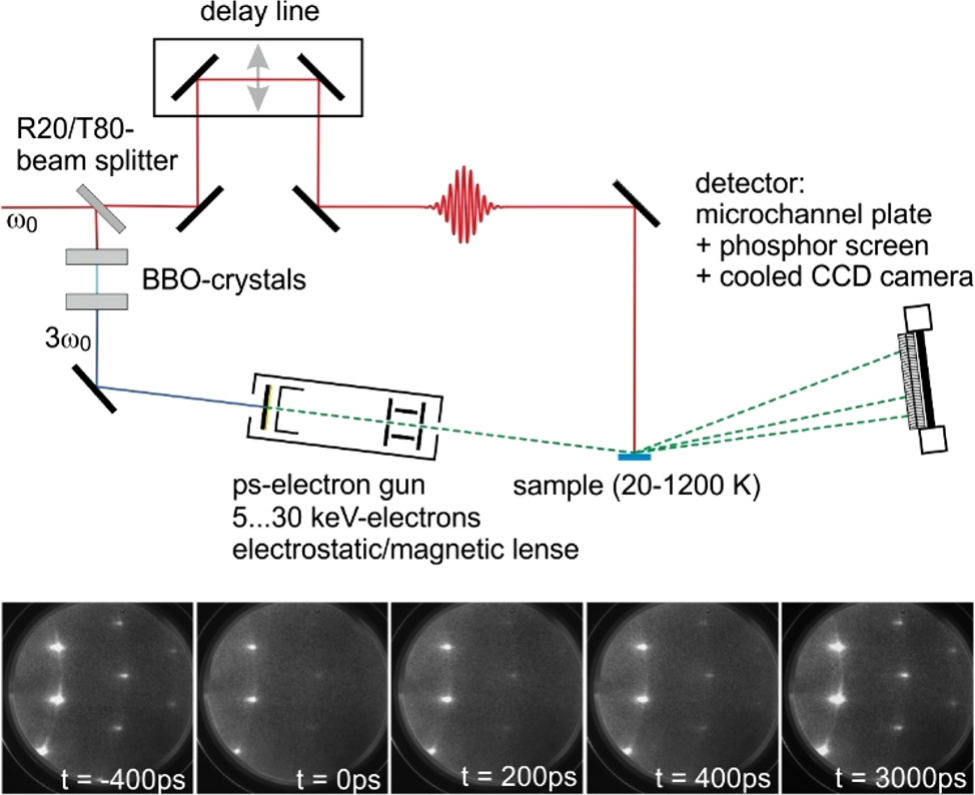
Sketch of the pump–probe setup of the ultrafast time-resolved RHEED experiment. The sample is excited by an infrared laser pulse. Part of the initial pulse is frequency tripled and generates the ultra-short electron pulse through single electron emission in a back-illuminated transparent photocathode. The electron pulse is accelerated to an energy of 5–30 keV and subsequently diffracted at the sample surface with grazing incidence. The time delay between the optical pump and the electron probe is varied by a mechanical delay line. The series of electron diffraction patterns of a Bi(111) film on Si(111) depicts snapshots of the transient intensity drop upon excitation with the fs-laser pulse and the recovery of intensity. Adapted with permission from Ref. 47.

Many processes on surfaces involving structural dynamics occur on their natural timescale within femto- to picoseconds. While optical spectroscopy or photo-electron spectroscopy can access these time scales, this was not such easy for diffraction techniques, which are the methods of choice for true sensitivity to ultrafast structural dynamics, i.e., determine changes in the geometric position of the atoms at the surface. The method of choice to accomplish this task is electron diffraction^1^ in a pump–probe setup, as it is sketched in Fig. 1. The surface is excited by an ultra-short laser pulse (pump), and the transient changes in an electron diffraction pattern are recorded after a time delay Δ*t* with an ultra-short electron pulse (probe). For negative delays, the sample is probed prior to the excitation and the ground state is accessible. With a systematic variation of the time delay, the transient response of the surface upon excitation could be determined and a movie of diffraction patterns as a function of Δ*t* was recorded. Such a movie is shown at the bottom panel of Fig. 1, with snapshots of the diffraction pattern of a 6-nm-thick epitaxial Bi film on Si prior to the excitation and after the excitation for various time delays. The electron pulse is generated from a photo cathode through photo emission after frequency tripling of a small fraction of the pump laser pulse, which renders this technique free of any jitter.

Surface sensitivity is achieved using electrons for diffraction, which exhibits a scattering cross section that is 10^5^–10^7^ larger than that for x rays of comparable wavelength or energy.^2^ As electrons of ∼50 eV exhibit an inelastic mean free path λ_mfp_ of a few Ångstrom only,^3,4^ they are employed in low-energy electron diffraction (LEED) as the standard technique for structural characterization in surface science. The atomic positions of the atoms in the surface unit cell can be determined to less than one tens of an Angstrom.^5–9^ However, such low-energy electrons are subject to various effects of severe temporal broadening of a photo-generated electron pulse. The broadening originates from the initial energy spread of the photoexcited electrons, low-field strength in the acceleration regime between photo cathode and anode, and space charge repulsion at higher numbers of electrons per pulse.^10–13^ To avoid such obstacles, the pioneers of such studies at surfaces used high-energy electrons at 25 keV.^10,14–17^ As the mean free path increases proportional to $\sqrt{E}$ with increasing energy *E,* we obtain λ_mfp_ between 2 and 5 nm.^3,4^ Surface sensitivity is then only obtained by grazing incidence of the electrons between 6° and 2°, i.e., in a RHEED geometry.

The practicability of such an experiment was demonstrated by early work of Elsayed-Ali *et al.* with sub-nano-second temporal resolution.^10,16^ Aeschlimann *et al.* improved the temporal resolution to a few tenths of picoseconds.^17^ Employing the Debye–Waller effect in diffraction, they studied superheating and premelting of surfaces of lead,^18–20^ bismuth,^21^ platinum,^17^ germanium,^22^ and indium.^23^ They also studied the dynamics of driven phase transitions on the Ge(111) surface.^23,24^ Zewail *et al.* were able to improve the temporal resolution to below 10 ps for seminal studies on interfacial water^25^ and adsorbates at surfaces.^26,27^

With the pioneering experiment by Siwick *et al.,*^28^ transmission electron diffraction (TED) through electron-transparent thin solid samples has achieved a temporal resolution well in the femtosecond regime. Since then, further improvement was possible through the optimization of the gun geometry^29,30^ or implementation of a pulse compression scheme by an RF cavity^31^ or THz manipulation.^32^ An overview of the still ongoing rapid development of this area of TED can be found in several review articles.^33–37^

The temporal resolution for the URHEED experiments, however, was still limited to the 10 pikoseconds regime through the so-called velocity mismatch^38,39^ between probing electrons at grazing incidence and pumping laser pulse at normal incidence. Baum and Zewail achieved a major breakthrough in temporal resolution by utilizing a tilted-pulse-front scheme for the pumping laser pulse, which boosted the temporal resolution in URHEED to only a few hundred femtoseconds.^40,41^

It is worth noting that Ropers and coworkers in a seminal work bypassed the problem of severe temporal broadening in ULEED through extreme miniaturization of the entire ULEED setup and succeeded to obtain an optimum temporal resolution of 1 ps.^42,43^ Applying spot profile analysis, they traced the phase ordering of a charge density wave the surface of 1T-TaS_2_ after optical excitation.^43,44^ An impressive example of the possibilities provided by ULEED is the coherent control of a surface structural phase transition employing a pump–pump–probe experiment.^45,46^

Here, we describe the setup of our URHEED experiment and demonstrate with a few examples of our research the huge potential of this technique for studying transient phenomena that are far away from equilibrium at surfaces.

## II. METHODS AND FUNDAMENTALS

### A. Experimental

**FIG. 2.**
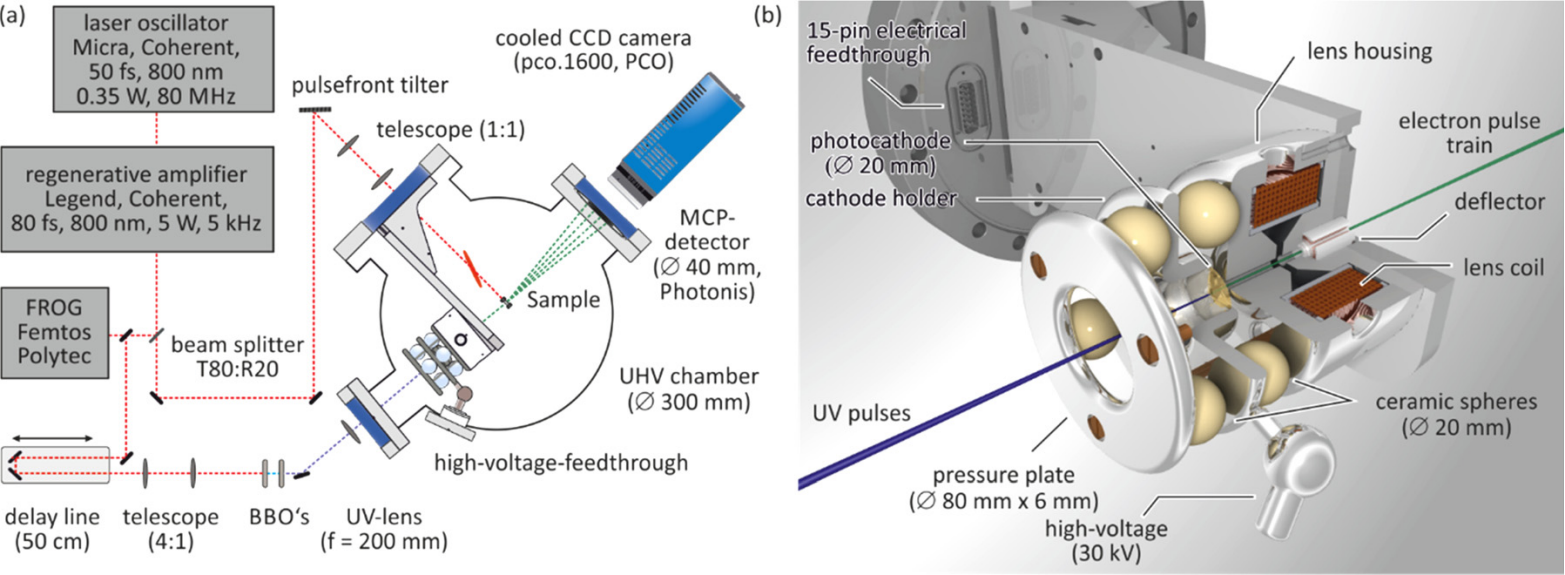
(a) Experimental setup of the time-resolved RHEED experiment. A horizontal cut through the UHV chamber shows the fs-electron gun, the sample position, and the detector unit. The paths of the electron pulse and diffracted electrons are shown as dashed green lines. The laser system and pulse front tilting are sketched. The pump laser path is shown as dashed red line. (b) Rendered image of the third-generation fs-electron gun. The blue and green beam are the UV-pulse and electron pulse, respectively. The ceramic spheres insulate the high voltage of the photocathode holder. Magnetic lens, magnetic deflectors, and HV-feedthrough are also shown. Adapted with permission from Ref. 49.

All experiments were performed under ultrahigh vacuum (UHV) conditions with a background pressure of less than 2 × 10^−10^ mbar in one single UHV chamber as sketched in Fig. 2(a). In addition to the RHEED setup, the UHV chamber was also equipped with a conventional low-energy electron diffraction (LEED) instrument for sample inspection and control of sample preparation.

Silicon samples measuring 16 × 2 × 0.5 mm^3^ were introduced through a load-lock system for easy sample exchange. The samples were mounted on a piezo motor-driven rotatable sample stage for adjusting the azimuthal angle. The sample stage was connected to a cryostat, which allows cooling to 90 K with LN_2_ and 20 K with He. The silicon samples could be heated up to 1400 °C by applying direct current heating. The manipulator allows motion with three degrees of freedom in translation and a second axis of rotation for the precise adjustment of the diffraction condition.

Si samples were prepared by degassing at 600 °C, followed by a short flash annealing close to the melting point, removing the native oxide. The deposition of adsorbates and the growth of epitaxial thin films were achieved *insitu* through molecular beams from small Knudsen cell or e-beam evaporators in the same chamber.^48^ Refreshing the surfaces of the samples through removal of adsorbates originating from residual gas was possible through repeated moderate re-heating with subsequent cooling.

The sample is excited through a regenerative titanium–sapphire laser amplifier at a repetition rate of 5 kHz, providing infrared light with a wavelength of 800 nm, i.e., a photon energy of 1.55 eV. The pulse duration is 50–80 fs with a pulse energy of 1 mJ. The pulse energy has to be high enough to provide homogeneous excitation of a sample area larger than the area that is probed by the electron beam. Due to the grazing incidence of the electron beam probes together with a width of the sample of 2 mm, the pumping laser pulse should not be focused to less than 4 mm in diameter. With our setup, we achieve an incident laser fluence up to 14 mJ/cm^2^.

With a beam splitter, a small part of the laser pulse is split off and frequency tripled in two barium borate crystals. The UV-pulse of 4.65 eV passes a MgF_2_ window and is guided to the fs-electron gun, which is shown in Fig. 2(b). The UV-pulse generates a fs-electron pulse through single-photon photoemission from a back-illuminated photocathode.^15,49–52^ The number of electrons in the pulse can be adjusted by the fluence of the UV-pulse through rotation of the λ/2 waveplate in front of the BBO crystals. The electrons are accelerated in a high extraction field of 7.5 kV/mm between the cathode and the anode to a kinetic energy of 30 keV. Space charge broadening and vacuum dispersion of the electron pulse require the use of fast electrons, here traveling at 1/3 of the speed of light. The larger the extraction field and the higher the final electron energy, the smaller the temporal broadening due to the initial energy spread Δ*E* due to photoemission and space charge broadening effects.^53^ The initial energy spread is only Δ*E* = 0.1 eV due to the use of a 10-nm-thick gold photocathode, where the work function of the Au film nicely matches the energy of the UV photons (3*h*ν = 4.65 eV).^51^

Subsequently, the electron pulse is focused by a magnetic lens located between anode and sample. At the exit of the magnetic lens, a x-y deflector has been integrated which is composed of two split pair coils allowing us to deflect the electron beam can by ±4° in order to change the electrons incident angle on the sample. The electron pulses are scattered under a grazing angle of 1°–6° at the sample, which is placed 50 mm beyond the guns exit aperture. The diffracted electrons are amplified through a micro-channel plate and recorded by a cooled CCD camera.

**FIG. 3.**
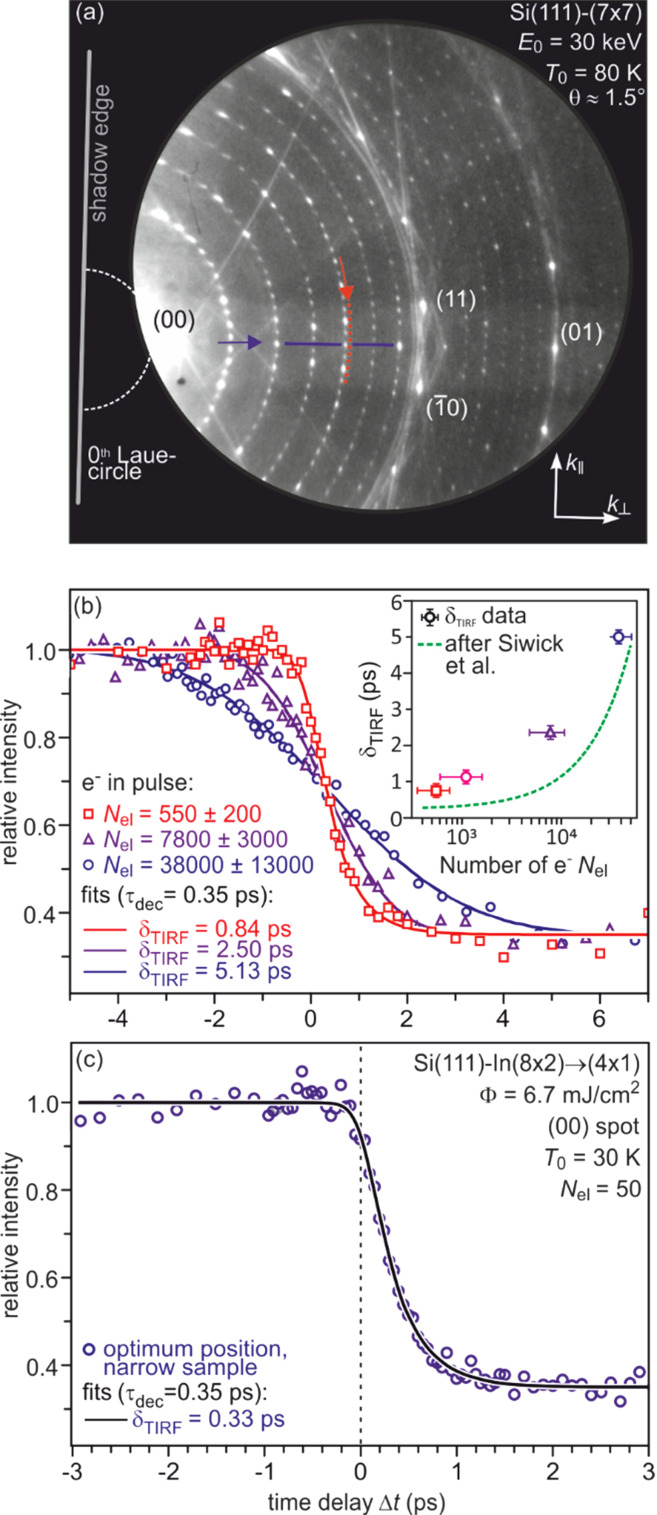
(a) shows a diffraction pattern obtained a 80 K from the bare Si(111) surface with its inherent (7 × 7) reconstruction. It is characterized for a large number of sharp diffraction spots and a low background intensity. In RHEED, the diffraction spots are organized on so-called Laue circles,^57^ which are centered at the shadow edge beneath the (00) spot, i.e., the specular reflected spot. (b) Temporal instrumental response function for various numbers of electrons per pulse. The inset shows a comparison of the FWHM with a simulation by Siwick *et al.*^53^ (c) Highest possible temporal resolution of FWHM = 330 fs for a narrow sample and low number of electrons per pulse. Adapted with permission from Ref. 49.

Along the Laue circles, the width of the diffraction spots amounts to 9% of the Brillouin zone, which is equal to 0.17 Å^−1^. Perpendicular to the Laue circles, here the 3/7 circle, the FWHM is only 0.65% of the Brillouin zone, which is equal to 0.012 Å^−1^. The transfer width is obtained by the reciprocal of the k-space resolution and amounts to *w*_trans,ǁ_ = 50 Å and *w*_trans,⊥_ = 550 Å along and perpendicular to the Laue circle, respectively. This large difference in the resolving power is characteristic for RHEED: the direction along the electron beam path is the high-resolution direction. The outstanding performance of the RHEED gun is clearly shown in the diffraction pattern in Fig. 3(a), which is taken from a Si(111)–(7 × 7) surface; all superstructure spots are clearly resolved against the low background. The high transfer width enables ultrafast spot profile analysis as will be demonstrated in Sec. III B.

The grazing incidence of the electrons causes severe velocity mismatch with the laser pump pulse under normal incidence.^38,39^ Though electrons of 30 keV travel already at a speed of 1/3 of light, they still need 20 ps to traverse a sample of a typical width of 2 mm. Over this long time, the transient intensity changes in the RHEED pattern are averaged, which is disastrous for the temporal resolution! Tilting the pump-pulse intensity fronts by 71° with respect to their propagation direction, a constant time delay between pump and probe pulse can be achieved.^40,41,54,55^ The pump-pulse front tilting is achieved by first-order back diffraction through a sinusoidal grating in (almost) Littrow geometry. Using a 1:1 telescope, the grating is imaged onto the sample. As a result, we obtain a tilted pump-pulse front at the sample with the desired tilting angle of 71°.^56^ Now, the resulting width of the overall temporal response function is ultimately given by electron and laser pulse widths.

An upper limit for the temporal resolution of our RHEED experiment has been determined from the transient changes of spot intensity during the structural response of an optically driven phase transition in the Si(111)–(8 × 2)↔(4 × 1) surface CDW system (we refer to Sec. III E). In Fig. 3(b), the number of electrons has been varied from 50 to 38 000 electrons per pulse. For pulses with a high-electron number *N*_el_, the temporal response becomes sigmoidal. Reducing *N*_el_ leads to an asymmetric temporal behavior around delay zero. By fitting the above function to the data, the FWHM of the temporal instrumental response function is extracted and displayed in Fig. 3(b) with a logarithmic scale of *N*_el_. The dashed green line in the inset of Fig. 3(b) depicts the result obtained by an analytic model for electron packet propagation developed by Siwick *et al.*^53^ The ultimate temporal resolution of 330 fs has been achieved for 50 electrons per pulse, which is only slightly larger than the theoretical achievable temporal resolution of 275 fs.^49^

The necessity of using electrons with high energy prohibits a normal incidence of the electrons for the diffraction experiment. With a dominant forward scattering and a mean free path of the order of ∼5 nm, the electrons are no longer surface sensitive. The large vertical momentum transfer for backscattering under normal incidence at such high-electron energies *E* = 30 keV would give rise to a huge Debye–Waller effect, i.e., would render a diffraction pattern impossible.

Surface sensitivity is achieved using a grazing incidence angle between 1° and 6° in a RHEED geometry. Then, the vertical momentum transfer

$$\Delta k_{⊥}=4\pi \;\;\text{sin}\;\;\theta \;/\lambda_{el}$$ (1)

is of the same order as in a typical LEED setup, i.e., for *E* = 30 keV energy at a typical grazing angle θ = 2.5°, we obtain Δ*k*_⊥_ ≅ 7.8 Å^−1^, which corresponds to *E* ≅ 57 eV at normal incidence in a LEED experiment—thus similar low penetration depth of a few Angstrom only.

The RHEED pattern is amplified with a multichannel plate and recorded from a phosphorus screen with a cooled CCD camera. To obtain a movie of RHEED patterns with varying time delays Δt, the arrival time of the pump laser pulse is changed through an mechanical delay line with a length of 2 × 50 cm^2^. This accounts for a possible range for the time delay of Δ*t* = −300 … 3000 ps.

Averaging and recording a typical RHEED pattern takes 10 s. In order to account for drift effects and slowly varying intensity fluctuations of the laser, each diffraction pattern is normalized by a pattern recorded without laser excitation of the sample. Obtaining an entire movie with 400 frames takes about 2 h.

### B. Spot position in diffraction

**FIG. 4.**
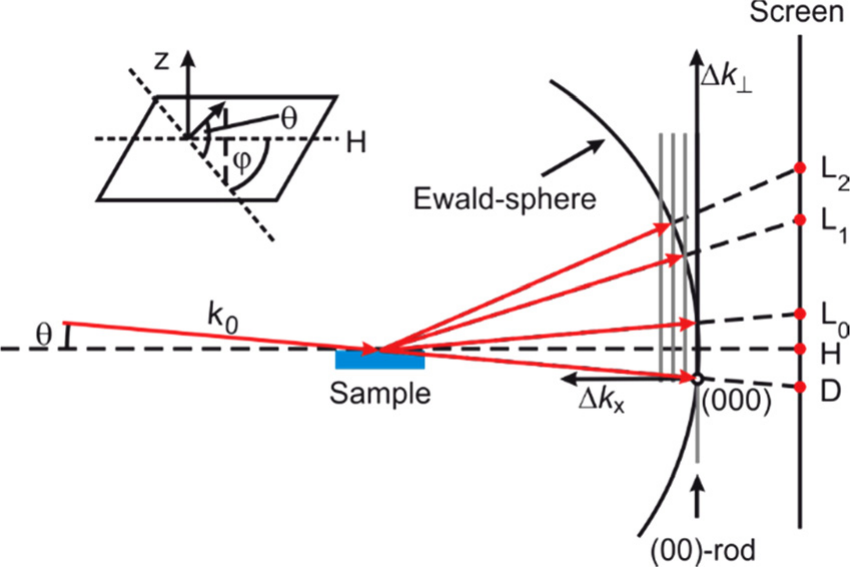
RHEED geometry. The electron beam is incident with a grazing angle θ. The position of the diffraction spots on the screen is determined by the intersection of the reciprocal lattice rods with the Ewald sphere. Adapted with permission from Ref. 58.

In general, the position of the diffraction spots is determined by the intersection of the Ewald sphere with the vertical lattice rods^57,59^ originating from the two-dimensional surface lattice as sketched in Fig. 4. Because the Ewald sphere and the reciprocal lattice rods intersect under grazing angle, the diffraction pattern is strongly distorted as compared to LEED. The RHEED spots are then located on the so-called Laue circles.

In a stationary experiment, the position of all spots—except for the (00) spot—varies as a function of temperature, thus reflecting the thermal expansion of the lattice. Such a shift of spot positions, however, could not been observed in an ultrafast RHEED experiment in the case of a flat surface. As already stated, the spot position is determined by the intersection of reciprocal lattice rods with the Ewald sphere. The location of the rods in reciprocal space is solely determined by the lateral lattice parameter of the surface. A sample with a macroscopic width *b* can expand laterally not faster than the speed of sound *c*_s_. A change of the lateral lattice parameter *a*_ǁ_ may then be expected for times *t* ≥ *b*/*c*_s_. For typical values of *b* = 1 mm and *c*_s_ = 10^4^ m/s, the lateral expansion of the lattice will take at least 100 ns, i.e., much longer than any delay time that can be accomplished by an optical delay stage of a reasonable length! The sample needs an extremely long time to react on the excitation by macroscopic lateral thermal expansion.

It is important to note that an expansion of the vertical lattice parameter, i.e., the distance between an adsorbate layer and the substrate, may occur as fast as a picosecond. A variation of this parameter, however, has no influence on the spot position in RHEED (or LEED). Of course, the intensity of the spots will be affected due to changes of the dynamic form factor, which is caused by the change of the unit cell geometry.^57^ This can easily result in large intensity variations along a rocking curve, i.e., significant intensity modulation along Δ*k*_⊥_. The scattering efficiency can then show rather sharp maxima in relation to the angle of incidence.^60,61^ If the spread of the incident electron angle is larger than the features in the rocking curve, the diffracted spot would indeed move when the features of the rocking curve change with the optical excitation.^62^ A direct measurement of the layer separation from such transient shifts of spot position is, however, not easily possible with RHEED.

Shifts of spot positions can also arise either from the lateral expansion of small islands of a rough surface or by space charge deflection in front of the sample.^26,63–66^ This space charge is usually generated by nonlinear photoemission induced by the intense pumping laser pulse. Such nonlinear effects are greatly enhanced for rough surfaces^67^ and higher laser fluences and should be interpreted with care. In our experiments, however, we never observed transient spot shifts up to the highest incident laser fluences of Φ ∼14 mJ/cm^2^.

### C. Debye–Waller effect in diffraction

The intensity of diffraction spots is attenuated by the thermal motion of the atoms as originally described by Peter Debye and Ivar Waller.^68,69^ The long-range translational order of a crystal is conserved upon heating up, and thus, sharp Bragg spots are principally present at all temperatures; however, it may be very weak. The short-range order, however, is gradually reduced with increasing temperature due to the enhanced thermal motion of the atoms, which causes loss of intensity from the sharp Bragg spots toward diffuse intensity. This behavior is described by the Debye–Waller effect^68,69^ in diffraction with the Debye–Waller factor (DWF)

$$I/I_{0}={\left\langle e^{i\Delta k\cdot u}\right\rangle }^{2}$$ (2)

with the normalized intensity *I*/*I*_0_ of the Bragg spot, Δ**k** the momentum transfer for this Bragg spot, **u** the vibrational amplitude of the atoms, and ⟨ ⟩ denoting time averaging. Assuming harmonicity of the atomic potentials in the material under study the DWF takes the form

$$I/I_{0}=e^{-\left\langle {\left[\Delta k\cdot u\right]}^{2}\right\rangle }.$$ (3)

Historically, the exponent is abbreviated by 2*M* = ⟨Δ**k⋅u**⟩^2^. The scalar product of Δ**k** and **u** can be employed to determine non-isotropic displacements through analysis of the Debye–Waller effect for different Bragg spots. While for surface-sensitive diffraction like LEED or RHEED, the vertical momentum transfer Δ*k*_⊥_ typically is much larger than the parallel momentum transfers Δ*k*_x_ and Δ*k*_y_ and these techniques are mostly sensitive to the vertical component *u*_⊥_ of the surface atoms displacements.

Assuming also isotropy of the harmonic potential, one may write

$$I/I_{0}=e^{-\frac{1}{3}{\;\Delta k}^{2}\left\langle u^{2}\right\rangle }$$ (4)

with ⟨*u*^2^⟩ the mean squared displacement (MSD). Inverting Eq. (4) provides access to the change of MSD upon excitation of lattice motion.

Within the Debye model of phonons, the square of the isotropic vibrational amplitude ⟨*u*^2^⟩ can be expressed by the temperature *T*, the mass of the atoms *m,* and the Debye temperature Θ_D_,

$$\left\langle {\vec{u}}^{2}\right\rangle =3\frac{3\hbar^{2}}{mk_{B}\Theta_{D}}\underset{\delta \left(T/\Theta_{D}\right)}{\underset{︸}{\left[\frac{1}{4}+{\left(\frac{T}{\Theta_{D}}\right)}^{2}\varphi \left(\frac{\Theta_{D}}{T}\right)\right]}}$$ (5)

with the normalized vibrational amplitude δ(*T*/Θ_D_) as function of the reduced temperature *T*/Θ_D_. The prefactor 3 arises from the three phonon branches, i.e., two transversal and one longitudinal branch. The integral φ(Θ_D_/T)

$$\varphi \left(\frac{\Theta_{D}}{T}\right)=\int_{0}^{\Theta_{D}/T}dx\;\frac{x}{e^{x}-1}$$ (6)

does not have an analytic solution. The temperature dependence of δ(*T*/Θ_D_) can, however, be approximated for low and high temperatures with sufficient accuracy. For *T* > Θ_D,_ the normalized displacements ε(*T*/Θ_D_) asymptotically reached *T*/Θ_D_. For this high-temperature range, Eq. (5) is simplified to

$$\left\langle {\vec{u}}^{2}\right\rangle =\frac{9\hbar^{2}T}{mk_{B}\Theta_{D}^{2}}.$$ (7)

In order to also consider the presence of zero-point fluctuations of the atoms for *T* < Θ_D,_ we have to use an alternative approximation for the MSD

$$\left\langle u^{2}\right\rangle =\frac{9\hbar^{2}}{mk_{B}\Theta_{D}}\left[\frac{1}{4}+{\frac{\pi^{2}}{6}\left(\frac{T}{\Theta_{D}}\right)}^{2}\cdot \left(1-e^{-\left(\frac{\pi^{2}}{6}-1\right)\frac{\Theta_{D}}{T}}\right)\right]$$ (8)

with a systematic deviation of less than 5% at higher temperatures *T* > Θ_D_.^70^

In addition, we also want to include the classical description within the Einstein model, in which independent harmonic oscillators with the frequency ω_cl_ are considered. Applying Boltzmann's equipartition theorem for a 3D potential, we obtain an average energy

$$\left\langle E\right\rangle =\frac{3}{2}k_{B}T=\frac{1}{2}\;m\omega_{\text{cl}}^{2}{\left\langle u^{2}\right\rangle }_{\text{cl}}.$$ (9)

In analogy to the Debye temperature Θ_D,_ we can define Θ_cl_ = *ℏ*ω_cl_/*k_B_* and find the MSD in the classical Einstein model

$${\left\langle u^{2}\right\rangle }_{\text{cl}}=\frac{3\hbar^{2}T}{mk_{B}\Theta_{\text{cl}}^{2}},$$ (10)

which is only valid for *T* > Θ_D_.

Since the Debye model provides a much more consistent description of the MSD as a function of temperature than the classic Einstein model, we will apply it throughout this review. The later is quite often used in the literature and thus needs to be acknowledged. A direct comparison of the two models reveals that the characteristic material-specific temperatures Θ_D_ and Θ_cl_ differ from each other by a constant factor of $\sqrt{3}$. This fact needs to be considered when determining and comparing Debye temperatures!

Determination of a transient temperature *T*(*t*) from the change of diffraction intensity during heating and cooling of the lattice subsequent to an optical excitation is only possible after thermalization of the phonon system, which occurs on few ps to 100 ps timescale. For shorter timescales, a temperature of the lattice may not yet be defined! This seemingly disadvantage can, however, been employed as a feature to determine non-equilibrium situations during the various steps of lattice excitation.^71,72^

## III. RESULTS

In all of the following sections, we will focus on few examples of our research to demonstrate how versatile this technique is for studies of dynamic processes on the nano-, pico-, and femtosecond timescale in surface science and nanoscale physics.

### A. Debye–Waller effect during thin-film cooling: Bi films on Si substrates

**FIG. 5.**
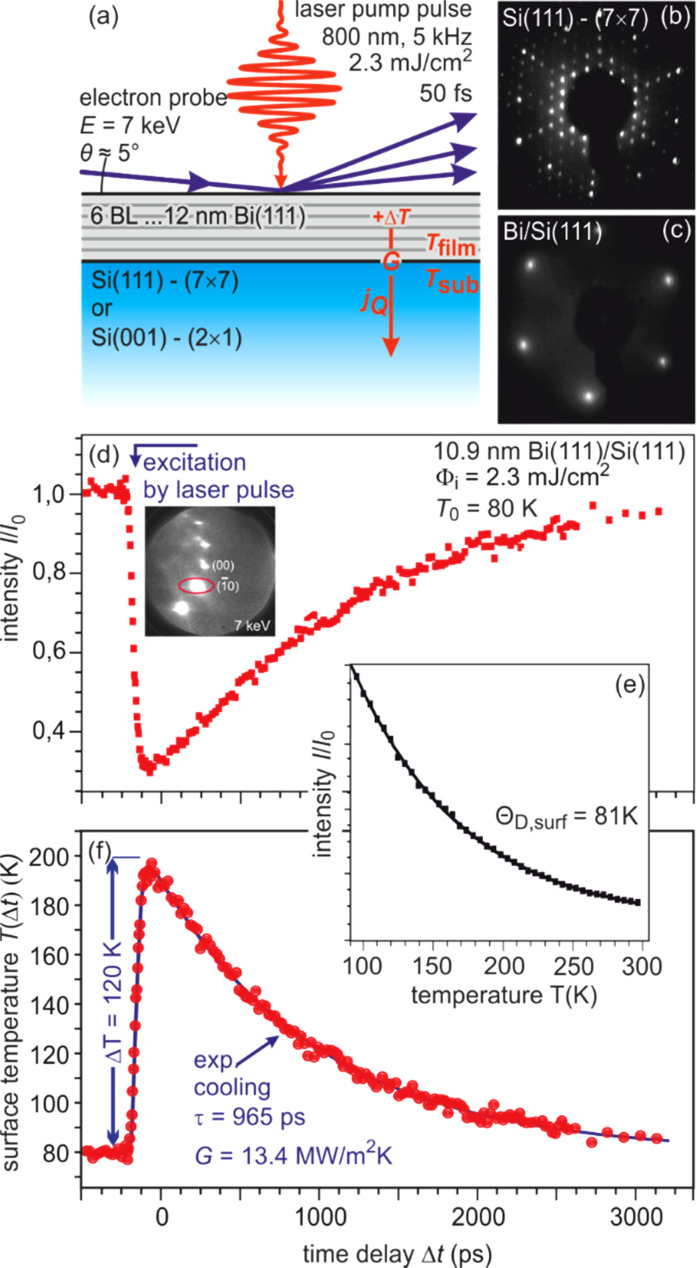
(a) Sketch of Bi–Si heterosystem. (b) LEED patterns of Si (111) and (001) substrates prior to and after growth of ultrathin Bi(111) films. (c) The intensity of the (00) spot is plotted as function of sample temperature during quasi-stationary heating. The intensity decrease is caused by the Debye–Waller effect. From the exponential slope, a surface Debye temperature Θ_D,surf_ = 81 K is derived. (d) The transient intensity drop of the (00) spot upon excitation with a fs-laser pulse at a fluence 2.3 mJ/cm^2^ at a sample temperature of *T*_0_ = 80 K exhibits an exponential recovery with a time constant of τ ≅ 1000 ps. (e) Using the stationary Debye–Waller curve taken under the very same diffraction conditions allows the direct conversion of the intensity drop to a transient temperature rise of Δ*T* = 120 K. The exponential cooling of the Bi film with τ_cool_ = 965 ps is determined through the thermal boundary conductance *G* = 13.4 MW/m^2^ K of the Bi–Si interface. Adapted with permission from Ref. 74.

One of the simplest experiments to demonstrate the proof of principle of time-resolved RHEED is the observation of transient heating and subsequent cooling of a single crystalline film on a substrate upon fs-laser irradiation. The transient decrease in spot intensity is interpreted by means of the Debye–Waller.^68,69^ For an isotropic thermal motion of the atoms, the attenuation of intensity is determined by Eq. (4) and allows to follow the transient temperature *T*(*t*) upon heating and cooling. As the vibrational amplitude *u* is inversely proportional to Θ_D_ [Eq. (7)], we expect higher sensitivity for surfaces with low Debye temperature Θ_D_. Therefore, we used thin films of bismuth with a bulk Debye temperature Θ_D,Bi_ = 112 K (Ref. 73), which were epitaxially grown on the surfaces of both Si(111)–(7 × 7) and Si(001)–(2 × 1) substrates as it is sketched in Fig. 5(a). The LEED patterns prior to and after growth of thin Bi(111) films are shown in the panels from Fig. 5(b). The films have been grown *in situ* by molecular beam epitaxy under UHV conditions.

The surface Debye temperature Θ_D,surf_ of the Bi(111) film has been determined from a stationary experiment where the sample was slowly heated from 90 to 320 K. The decrease in intensity of the (00) spot is plotted in Fig. 5(c). From the fit with an exponential function, we obtain Θ_D,surf_ = 81 K for the surface Debye temperature using the Debye model. This surface Debye temperature is somewhat lower than the Debye temperature of the bulk, as the surface atoms have fewer bonds and are therefore more weakly bound.

The fs-laser excitation with a fluence of 1.3 mJ/cm^2^ at a sample temperature of *T*_0_ = 80 K results in a pronounced intensity drop of more than 60% as shown in Fig. 5(d). Applying Θ_D,surf_ = 81 K and a vertical momentum transfer of Δ*k*_⊥_ = 6.7 Å^−1^ to Eqs. (2) and (3), this converts into a sudden jump in the surface lattice temperature of Δ*T* = 120 K up to *T*_0_ = 200 K. The recovery of the ground state occurs by an exponential cooling with a time constant of τ_cool_ = 965 ps, which is determined through the thermal boundary conductance *G* = 13.4 MW/m^2^ K of the Bi–Si interface.

Such a slow cooling rate is described in the framework of well-established models for heat transport: the acoustic mismatch model (AMM) and the diffuse mismatch model (DMM). The discontinuity of the elastic properties at the interface between the Bi(111) film and the Si(111) substrate gives rise to an additional thermal resistance first described by Kapitza.^75^ Under these conditions, the slow cooling of the Bi film can be described within the DMM.^76–78^ The energy is carried by phonons that were diffusively scattered at the Bi–Si interface. Applying Fermi's Golden Rule, the density of phonon states determines the final state, i.e., the transmission probability from Bi film into Si substrate. Due to the much lower Debye temperature of Bi in comparison with Si, most of the phonons remain in the Bi film and only 1 of 100 is transmitted to the Si substrate. This results in a drastic slowdown in the cooling of the Bi film.^79,80^

**FIG. 6.**
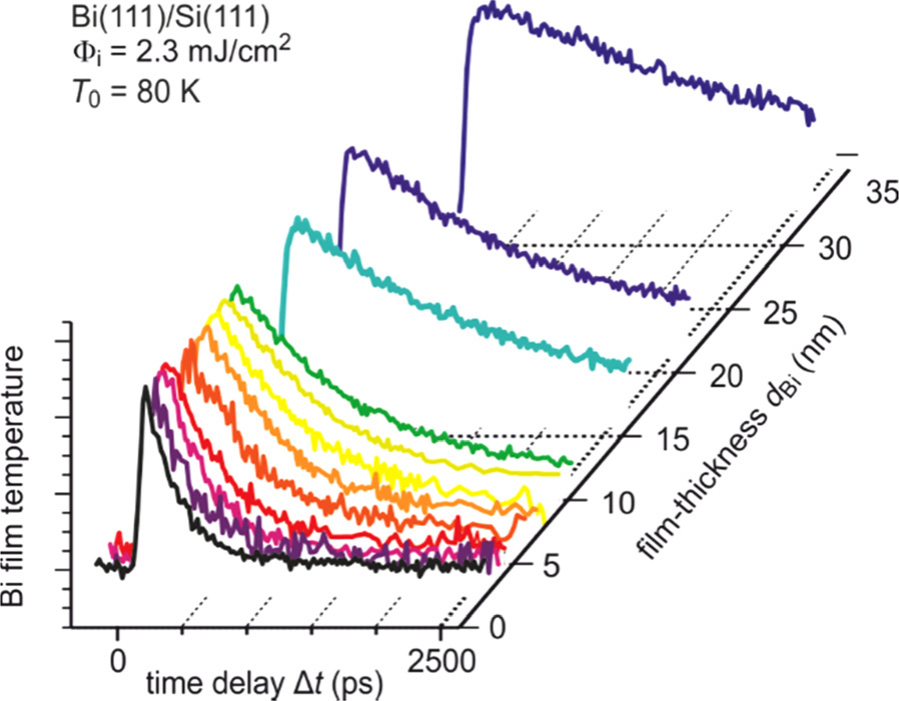
Evolution of temperature of Bi films with various thicknesses on Si(111) upon impulsive excitation at *t* = 0. The cooling time constant increases with film thickness. A mono-exponential recovery to the ground state is observed.

**FIG. 7.**
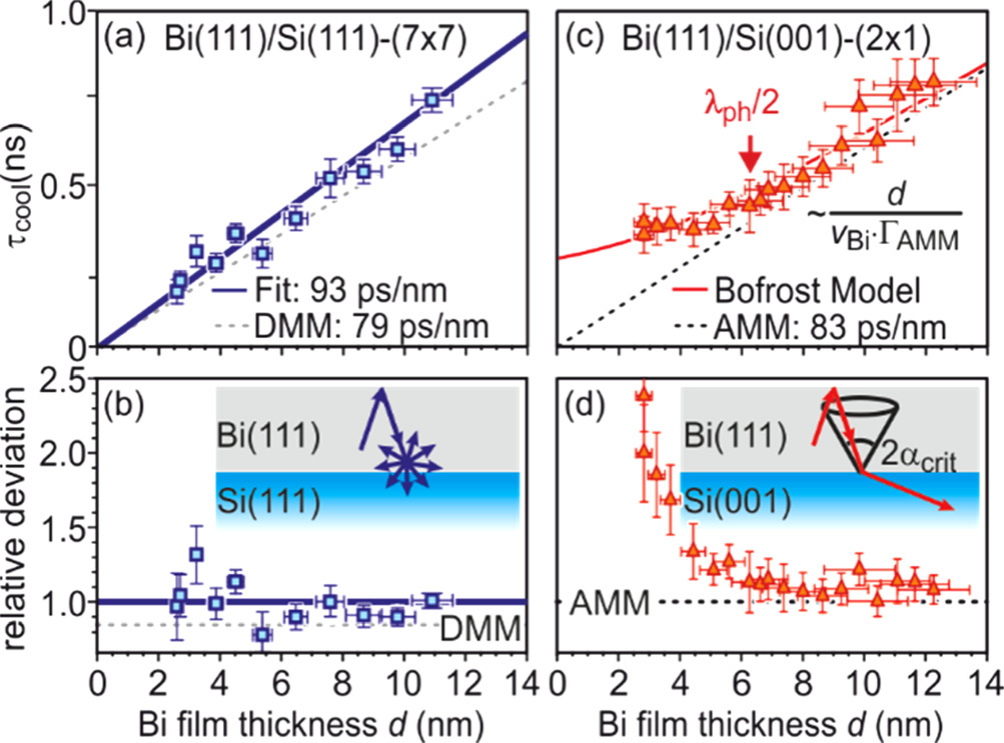
Thickness and substrate dependence of the cooling process. (a) Cooling time constant τ_cool_ as function of film thickness d of epitaxial Bi(111) films grown on Si(111). A linear dependence of τ_cool_ with *d* is observed. (b) Relative deviation of τ_cool_ from the linear fit shown in (a). The inset depicts the diffuse phonon scattering at the interface as expected from DMM. (c) Cooling time constant τ_cool_ of epitaxial Bi(111) films grown on Si(001). A deviation from the linear dependence of τ_cool_ with *d* is obvious for *d* < 6 nm. (d) Relative deviation of τ_cool_ from the linear behavior which is shown as dashed line in (c). The significant deviation below 6 nm is well described by our non-equipartition model (solid red line). The inset depicts the ballistic phonon transmission with refraction and reflection at the interface as expected from AMM. The critical cone of total internal reflection is sketched by dashed lines. In all experiments, the incident fluence of the 800 nm, 50 fs-laser pulses was set to 2.3 mJ/cm^2^, and the sample base temperature T_0_ = 80 K. Adapted with permission from Ref. 74.

The Bi film thickness dependence of the cooling time constant τ_cool_ gives insight into finite size effects in nanoscale heat transfer from ultrathin films across interfaces toward substrates. The evolution of transient temperature for such a thickness series is shown in Fig. 6 for various Bi(111) films epitaxially grown on Si(111) for 2.6 nm ≤ *d*_Bi_ ≤ 32 nm. The large variation of cooling time τ_cool_ for different film thicknesses is obvious and plotted in Fig. 7 as function of film thickness *d*_Bi_ for Bi(111) films grown both on Si(111) and Si(001) substrates.

Figures 7(a) and 7(b) show the behavior the Si(111) substrate in comparison with predictions from DMM. We observe the expected linear dependence of τ_cool_ ∼ *d*_Bi_. In contrast, Figs. 7(c) and 7(d) show a saturation of the cooling rate τ_cool_ for Bi films with *d*_Bi_ < 6 nm. To exclude islanding of the Bi(111) films grown on Si(001), which would give rise to a trivial explanation of the observed deviation, we confirmed that even the thinnest Bi film of 2.8 nm, i.e., only seven atomic Bi layers, is continuous and of homogeneous thickness. Such, it covers the entire sample and is not broken up (see Fig. S2 in supplementary material of Ref. 74). We attribute this astonishing deviation from the linear slope to a finite size effect, which occurs for the AMM for films thinner than half of the mean free path of the phonons, which for Bi is 12 nm at 80 K.

**FIG. 8.**
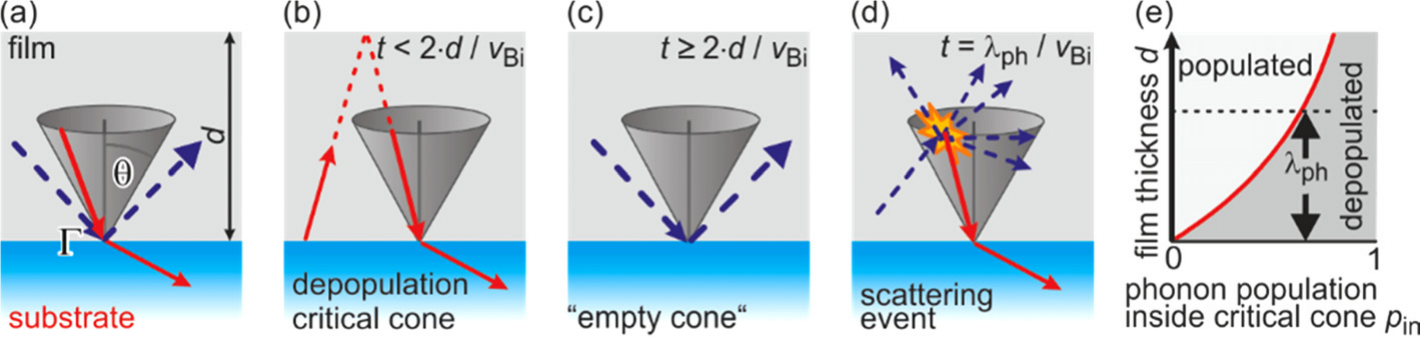
Non-equipartition model. (a) The vast differences in speed of sound in Bi and Si cause strong refraction of the phonons at the Bi–Si interface. Phonons in the Bi film with an incident angle smaller than the critical angle for total internal reflection—sketched as critical cone—overcome the interface, thus becoming refracted and transmitted into the Si substrate (red solid arrows). Phonons with an incident angle larger than the critical angle undergo total internal reflection and are trapped in the film (blue dashed arrows). The transmission probability Γ is defined as ratio of the energy between the transmitted waves compared to the incoming waves. (b) For ultrathin films, the phonons with incident angle inside the critical cone are depopulated after τ_depop_ > 2*d*/*v*_Bi_. (c) The critical cone is empty, cooling stops, and a pronounced non-equipartition between phonons inside and outside the cone has evolved. (d) Repopulation of the depopulated critical cone is possible through diffuse scattering events, e.g., umklapp and normal processes, after τ_therm_ = λ_ph_/*v*. Thermalization and repopulation of the critical cone are the bottleneck limiting cooling of the nanoscale film. (e) Non-equipartition of phonons inside the critical cone as a function of film thickness *d*. Adapted with permission from Ref. 74.

We explain this unexpected slowing down of the cooling rate in terms of a pronounced non-equilibrium distribution of phonons in angular phase space for films thinner than one half of the phonon mean free path λ_ph_. Within AMM, the heat transfer across an abrupt and smooth interface is determined by the reflection, transmission, and refraction of elastic waves following Snell's law, i.e., phonon momentum is conserved.^78,81,82^ In our model system as sketched in Fig. 8(a), the speed of sound in the Bi film (*v*_Bi,l_ = 1972 m/s, *v*_Bi,t_ = 1074 m/s) is much lower than in the Si substrate (*v*_Si,l_ = 8433 m/s, *v*_Si,t_ = 5845 m/s). Similar to the optical counterpart, total internal reflection of phonons occurs for incident angles larger than a critical angle α_crit_ = arcsin(*v*_Bi_/*v*_Si_). Only phonons with incident angles smaller than α_crit_, i.e., inside the critical cone, can overcome the hetero-interface as sketched in Fig. 8(b). Due to the almost perfect matching of the acoustic impedance of Si and Bi, all phonons inside the critical cone pass over to the Si substrate. Phonons outside the critical cone, however, undergo total internal reflection and stay trapped in the film.

Phonons inside the critical cone escape the Bi film after τ_depop_ = 2*d*/*v*_Bi_, which is the phonon transit time through the film considering specular backreflection at the surface as sketched in Fig. 8(c). For a 3-nm Bi film, the phonon phase space inside the critical cone is depopulated after τ_depop_ = 3 ps considering the speed of sound of the longitudinal phonon mode *v*_Bi_ = 1972 m/s as shown in Fig. 8(d), which is the most relevant for the heat transfer. After this time, the film would stop cooling since all other phonons are trapped in the film due to total internal reflection, following Snell's law!

Furthermore, cooling is only possible through scattering of phonons, which results in a repopulation of the critical cone as sketched in Fig. 8(e). This non-equilibrium situation is not covered by the simple AMM, which always assumes an equilibrium population between all phonon modes, i.e., the equipartition of the phonon system.^83,84^ This assumption is justified only for thicker films, where the transit time *d*/*v*_Bi_ is much longer than the scattering time τ_therm_ = λ_ph_/*v*_Bi_ of phonons.

In these films, the cooling time constant τ_cool_ is given by the transit time of a phonon through the film divided by the phonon transmission probability Γ across the interface: τ_cool_ = *d*/(*v*_Bi_⋅Γ).^74^ For films thinner than λ_ph_/2, equilibration and repopulation of the critical cone become the bottleneck for cooling. Then, normal and umklapp scattering processes among phonons are the dominant source for repopulation of the critical cone.

We now turn to the dissimilar behavior of the Bi films grown on Si(111), where a deviation from the equilibrium model is not observed in Figs. 7(a) and 7(b). Here, diffuse scattering at the interface has to maintain equipartition in the phonon system and governs the heat transfer. The bare Si(111) surface exhibits a (7 × 7) reconstruction, which consists of structural elements like staking faults, dimer rows, and corner holes, which are overgrown during low-temperature Bi deposition.^85^ All these structural elements are very likely sources for effective scattering of phonons and thus equilibrating different momentum states without changing their energy.^86^ The large unit cell of 2.7 × 2.7 nm^2^ size offers ample reciprocal lattice vectors for the necessary momentum transfer of diffuse elastic scattering of the phonons at the interface. This is different for Bi(111) films on the Si(001) surface with a (2 × 1) reconstruction consisting of dimers with a simpler and much smaller unit cell of 0.77 × 0.38 nm^2^ size.^74^

### B. Spot profile analysis of cooling of nanostructures: Ge clusters on Si(001)

Here, we employ nanoscale heat transport from self-organized germanium (Ge) nanostructure into a silicon (Si) substrate^87,88^ to demonstrate the capabilities of tr-RHEED to observe various transient processes in parallel from the same diffraction experiment. The standard techniques for the determination of the transient temperature evolution of thin films are ultrafast optical methods like time-domain thermoreflectance (TDTR).^89,90^ Due to the low scattering cross section of light with matter, they are, however, usually restricted to thicker films. Furthermore, the measured transient change of reflectivity is an integral response of the entire probed sample surface. Thus, it is not possible to distinguish between the transient response of different nanoscale structures, e.g., self-organized clusters of different dimensions, which may simultaneously be present at the sample. Here, we demonstrate in detail how time-resolved spot profile analysis in electron diffraction can be used to distinguish between the transient contributions from three different cluster types upon impulsive excitation by an intense fs-laser pulse.

**FIG. 9.**
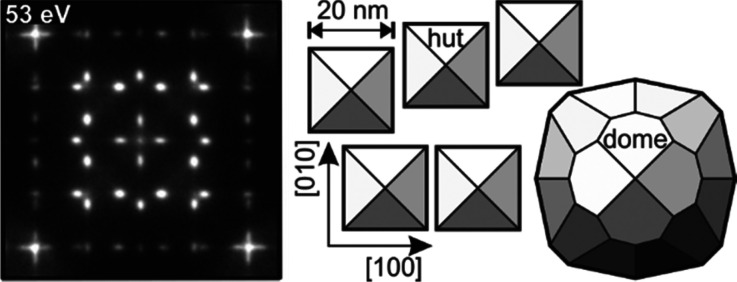
Illustration of the two different cluster types: hut clusters, composed of four {105}-type facets, and larger dome clusters with a complex faceting. The LEED pattern reflects the diffraction from the four {105}-type facets of the hut clusters. Adapted with permission from Ref. 88.

The self-organized growth of Ge on Si(001) was utilized to prepare a sample with well-defined epitaxial hut-, dome-, and relaxed Ge clusters, which were used as a model system. At typical growth temperatures between 400 and 700 °C, islanding of Ge is observed after the formation of a thin wetting layer, i.e., the so-called Stranski–Krastanov growth mode.^91^ Initially, a lattice matched Ge film grows in a layer-by-layer fashion with the lattice constant of the underlying Si substrate. The strain energy increases with film thickness. This wetting layer becomes unstable for thicknesses of more than three monolayers of Ge (1 ML = 6.24 × 10^14^ atoms/cm^2^). With further increasing Ge coverage, the formation of a metastable phase, so-called hut clusters, is observed, which is explained as a first step on the kinetic pathway from layer-by-layer growth to islanding.^92–94^ Hut clusters are composed of four {105}-type facets, which form an angle of 11.3° with the {001} Si substrate surface plane. This leads to a rectangular or square shape, as sketched in Fig. 9. Typical dimensions are 23 nm width and a height of only 2.3 nm.^94,95^ As soon as the whole surface is covered with hut clusters, which should typically be the case after about 6 ML—a transformation to the next step larger islands is observed.^96–98^ The formation of steeper facets allows for a more efficient reduction of lattice mismatch induced strain than in the case of huts.^98–100^ With respect to their special shape, these larger clusters are usually denoted as dome clusters. Due to their complex faceting structure, their base area exhibits an approximately round shape with a diameter of 50–60 nm at a height of 5–6 nm, as sketched in Fig. 8. Both cluster types are free of lattice mismatch relieving defects and dislocations.^101,102^ The build-up of strain and the subsequent strain relief by islanding is the driving force for this self-organized and kinetically self-limited formation of clusters. For this reason, their size distribution is very narrow.^102–105^ The strain is reduced during the different transition states from layers to huts and then to domes. Hut clusters are still lateral compressed, and the vertical layer distance Δ*d/d* = 4% is significantly expanded through tetragonal distortion.^97,98,101^ In the case of dome clusters, relaxation toward the Ge bulk lattice constant is more efficient.^106^ Finally, the generation of defects and dislocations accommodates the lattice mismatch and causes the formation of large and fully relaxed 3D islands. Such islands do not grow any longer in a self-organized and kinetically self-limited way. Consequently, those relaxed clusters exhibit a very broad size distribution.^102,107^

**FIG. 10.**
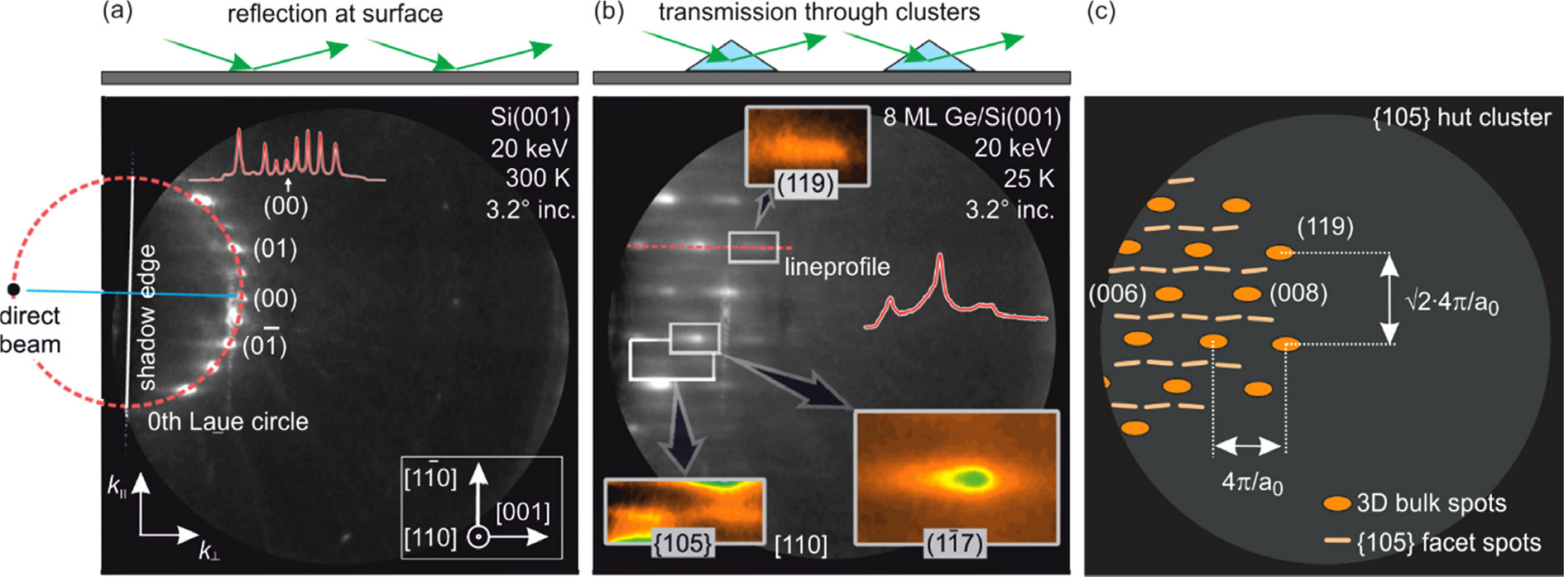
Electron diffraction patterns of (a) the bare Si(001) surface prior to deposition and (b) after deposition of 8 ML Ge, grown at 550 °C. The diffraction geometry was in both cases the same, i.e., the incidence angle was 3.2° at an electron energy of 20 keV. (c) shows a schematic diffraction pattern of a surface covered with {105} faceted hut clusters under the same diffraction conditions. The presence of regularly ordered spots instead of a circular arrangement indicates that the diffraction happens in transmission. Adapted with permission from Ref. 88.

Figure 10(a) shows the RHEED diffraction pattern of the initial bare Si(001) surface prior to deposition. Electrons were incident along the [110] direction. The grazing incidence of the electrons leads to a vertical penetration depth of a few Angstroms only. A series of intense spots show up located on the zero-order Laue circle, i.e., clear indication for diffraction from an atomically flat, single crystalline surface.

After deposition of 8 ML of Ge, a sample with a high coverage of hut clusters (8 × 10^10^ cm^−2^) and dome clusters (2 × 10^9^ cm^−2^) was prepared. The change of morphology directly affects the electron diffraction pattern, as can be seen in Fig. 10(b). The typical circular arrangement of the spots for reflective diffraction from a flat surface has changed into a periodic ordered arrangement of equidistant spots, which is indicative for diffraction in transmission geometry from single crystalline or epitaxial structures. This is in contrast to observations of Debye–Scherrer rings arising from diffraction from disordered, polycrystalline nanoparticles.^25^ Because the grazing angle of incidence of the electrons of 3.2° is much lower than the hut cluster facet angle of 11.3°, electrons cannot be diffracted in reflection geometry from the {105} facets with orientations along the incident electron beam. Instead, they have undergone diffraction in transmission through the clusters. Thus, the diffraction pattern is described by a cut through a reciprocal diamond lattice in [001] direction. As depicted in the schematic diffraction pattern in Fig. 10(c), the distance of the spots is then given by 4π/*a*_0_ in [001] direction and in [1-10] direction by √2·4π/*a*_0_, respectively, and *a*_0_ the size of the cubic unit cell.

With the knowledge of the position of the (00) spot of the Si(001) substrate [see Fig. 10(a)] and the grazing angle of incidence of 3.2°, we can assign each spot to a Bragg reflection for a diamond lattice. Kinematically forbidden spots are described in terms of double diffraction, which is a prevalent effect in RHEED.^57,108^ Figure 10(c) also shows the original position of the zero-order Laue circle. Thus, all Bragg reflections in the vicinity of this circle have a very low distance to the Ewald sphere, and hence, the corresponding spots exhibit a high diffraction intensity, which is clearly visible in Fig. 10(b).

In addition to the transmission spots, we also expect reflection spots from those {105} facets oriented perpendicular to the incident electron beam as reported by Aumann *et al.*^109^ Each of the transmission spots is then accompanied by four {105} facet surface diffraction spots facing toward the transmission spot. These facet spots are located between the transmission spots and can clearly be identified in the contrast enhanced inset of Fig. 10.

**FIG. 11.**
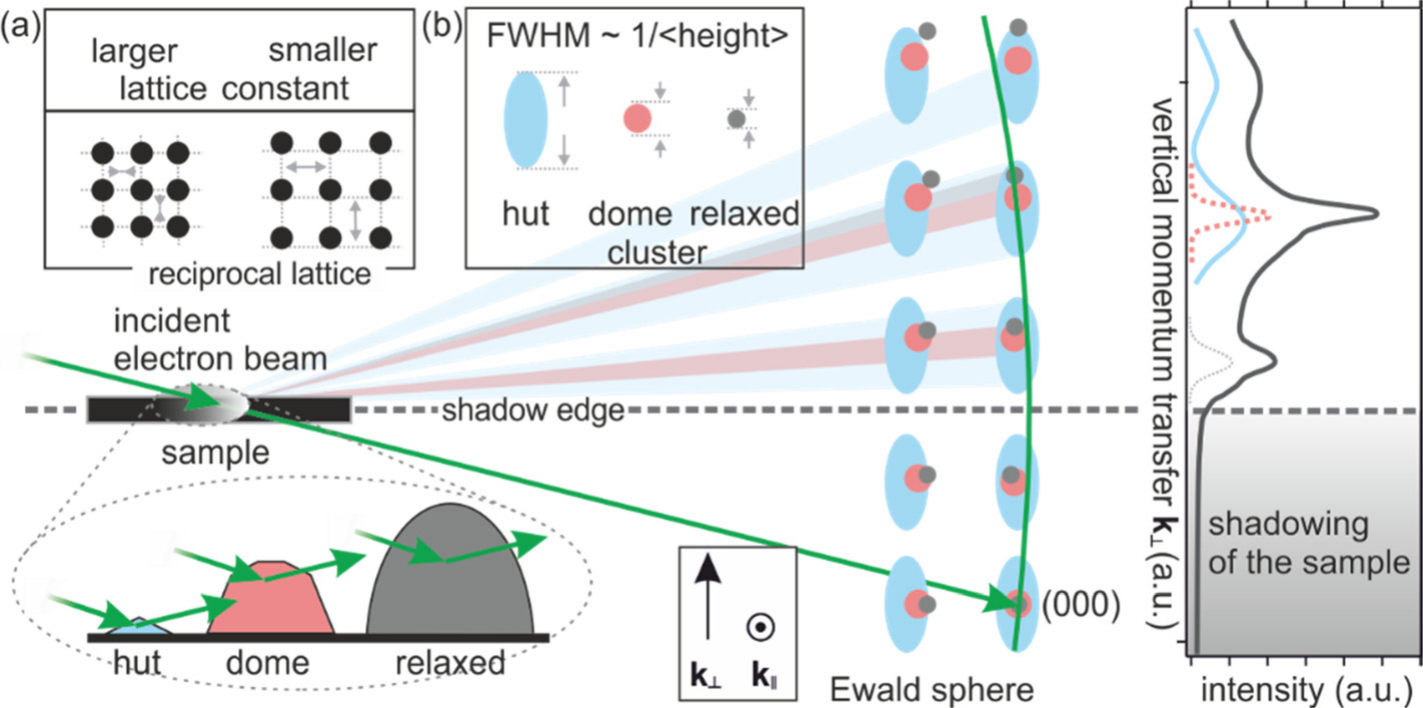
Sketch of transmission diffraction geometry and Bragg conditions in reciprocal space for hut (blue), dome (red), and relaxed clusters (gray). The Ewald sphere is plotted in green. The Bragg conditions are broadened due to finite size effects and are shifted vertically and laterally due to variations of strain state. For simplicity, a simple cubic lattice is used. (b) The spot profile depicts the variation of diffraction intensity of the (117) and (119) spots along the *k*_⊥_ direction [see also red dashed line in Fig. 10(b)]. The profile can be fitted by two broad and narrow Gaussians assigned to diffraction from huts and domes, respectively. Adapted with permission from Ref. 88.

In order to distinguish the contributions from the different cluster types to the diffraction pattern, we applied spot profile analysis. The right panel of Fig. 11 shows a line profile along the marked red line in Fig. 10(b). The intensity is plotted as function of *k*_⊥_ along the direction through the (117) and the (119) spot. The experimental data can be described by a pair of two Gaussians of different widths, positions, and intensities. The broadening and shift of spots is explained in terms of different size and strain state of the huts, domes, and relaxed clusters as is schematically depicted in the left panel of Fig. 11.

During diffraction, the finite size of the clusters effectively acts as a slit with finite width, i.e., the height, width, and length of the clusters. Thus, the diffraction spots of the smallest structures, i.e., the hut clusters, should exhibit a significant broadening in reciprocal space due to finite size effects. This broadening is most effective along the vertical momentum transfer *k*_⊥_ as the smallest dimension of the huts is their height, as sketched in the inset of Fig. 11(b). For the dome clusters, we expect narrow spots because they exhibit a height, which is 3 to 4 times larger than that of the huts. We therefore assign the broadened Gaussians [blue dotted line in Fig. 11(b)] to the 2 nm high hut clusters. The FWHM (full width at half maximum) of vertical momentum of 1.0 Å^−1^ is slightly larger than the value of 0.62 Å^−1^, which is expected for diffraction from the entire hut cluster, i.e., assuming infinite mean free path of the electrons, kinematic approximation, and volume weighted hut cluster profile in the direction parallel to the diffraction vector. The narrow Gaussian (red dashed line) originates from the higher and larger dome clusters.

The different strain states of the three cluster types result in different positions of the corresponding diffraction spots. Hut clusters are coherent to the Si substrate and exhibit a 4% increased layer separation by tetragonal distortion.^98^ This vertical strain causes a significant shift of the corresponding spots to lower values of Δ*k*_⊥_ = 62 Å^−1^, i.e., toward the (000) Bragg condition or the shadow edge, respectively. From the observed shift Δ*k*_⊥_ of the position of the broad Gaussian to lower values of Δ*k*_⊥,_ we conclude an increased layer separation of Δ*d/d* = 2.6% of the hut clusters with respect to the dome clusters since the apex of the dome clusters is more relaxed than hut clusters.^97,100^ The lateral compression of the hut clusters toward the Si lattice parameter can also be observed through a shift of the spot positions of the broad Gaussian away from the (00) rod to larger values of *k*_‖_, as sketched in Fig. 11.

Up to now, we have used the width and the position of the diffraction spots to distinguish between contributions from huts and domes. The intensities of these peaks are determined by the dynamical structure factor *F*_hkl_ and the density of the clusters on the sample, i.e., the diffraction volume, which both are not well-defined quantities. Last but not least, also the Laue condition **Δk **=** G** affects the relative intensities and may be used to distinguish between huts and domes. The Laue condition is fulfilled when reciprocal lattice points or lattice rods are intersected by the Ewald sphere, i.e., for spots on the zero-order Laue circle or in its direct vicinity as sketched in Fig. 11. In case that the reciprocal lattice points are broadened (due to finite size effects), this condition becomes relaxed. Therefore, diffraction from hut clusters (broad light blue spots in Fig. 11) is still observed for larger vertical momentum transfer, where the dome clusters (light pink spots) no longer contribute to the diffraction pattern because their reciprocal lattice spot is no longer intersected by the Ewald sphere (green line). Such a case is observed for the (119) spot, where the relative intensity of the narrow Gaussian is much smaller than for the (117) spot, which is located close to the zero-order Laue circle. Thus, all spots on the right-hand side of the diffraction pattern in Fig. 10(b) arise almost solely from hut clusters.

**FIG. 12.**
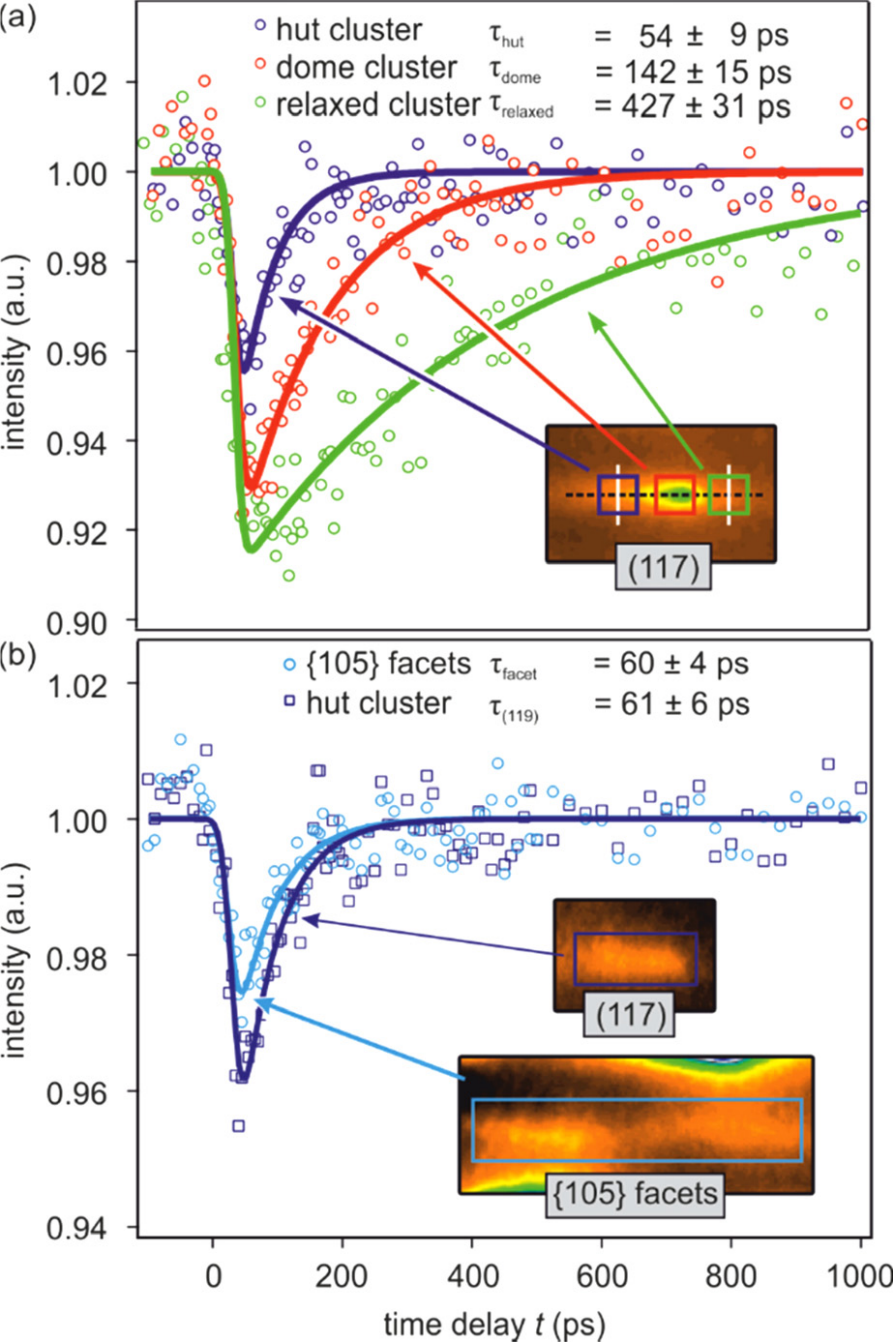
Transient diffraction intensities as function of time delay Δ*t* (a) different positions of the (117) spot exhibit three different recovery time constants which are assigned to diffraction from hut, dome, and relaxed clusters. (b) The transient intensity of the (119) spot and the {105} facet spots originate from hut clusters. Experimental data were fitted by a convolution of mono-exponential decay with a temporal response function of 25 ps width. Adapted with permission from Ref. 88.

The heating of the Ge clusters upon impulsive fs-laser excitation and subsequent cooling to the substrate are determined from the transient intensity drop, which is shown in Fig. 12 for different diffraction spots. Figure 12(a) shows the transient intensity of the (117) spot as function of time delay Δ*t* between the pump laser pulse and the probe electron pulse. The initial drop at the temporal overlap Δ*t* = 0 reflects the heating from 25 to 150 K. Applying a stationary intensity vs sample temperature curve for calibration of the Debye–Waller effect, we were able to determine the transient temperature evolution and the maximum temperature rise subsequent to fs-laser excitation, which is known to happen on a picosecond timescale.^110^ Here, the observed timescale of 25 ps is determined by the temporal response function of the RHEED setup, which was at that time still dominated by the velocity mismatch between pumping laser pulse and probing electron pulse.^47^ The recovery of the spot intensity occurs on a slower timescale and reflects the cooling of the Ge hut clusters through heat transfer to the Si substrate. Applying the above-described spot profile analysis, the contributions from hut-, dome-, and relaxed clusters could be discriminated from the profile of the (117) spot. The solid lines are fits to the data assuming an exponential recovery of intensity.

We observe three distinct different recovery time constants of τ_hut_ = 54 ps, τ_dome_ = 140 ps, and τ_relax_ = 430 ps, which were assigned to hut, dome, and relaxed clusters, respectively. The results from the transient spot profile analysis are supported by an independent analysis of the (119) spot and the {105} facet spots, which are shown in Fig. 12(b). The intensity of the (119) spot (open squares) is dominated by diffraction from hut clusters and reveals a recovery with τ_(119)_ = 61 ps. The {105} facet spots arise solely from the hut clusters and recover on a timescale of τ_facet_ = 60 ps. All three recovery time constants for the hut clusters are in good agreement, and we obtain an average cooling time constant of τ_hut_ = 58 ps.

**FIG. 13.**
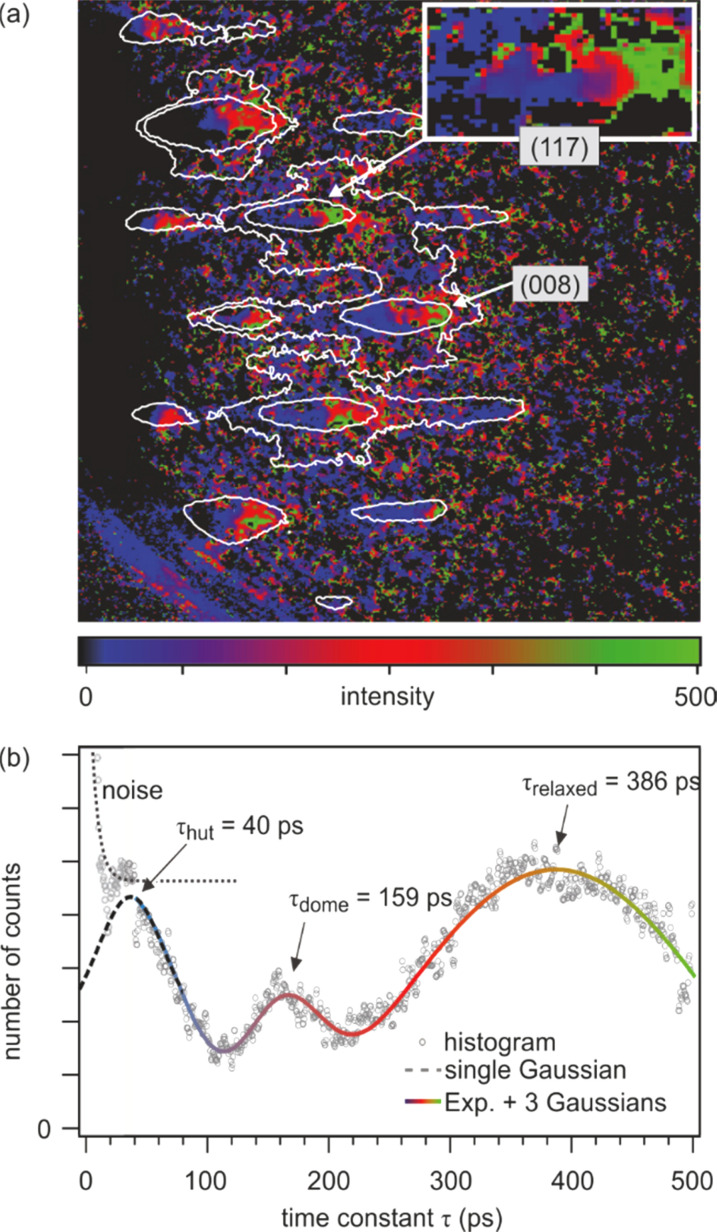
Pixel-by-pixel analysis of the entire diffraction pattern, the so-called lifetime map. (a) Color separation of diffraction spots indicates different cooling time constants for hut, dome, and relaxed clusters. (b) The histogram of the lifetime map analysis clearly shows three distinct cooling time constants, which agree well with the transient spot profile analysis depicted in Fig. 11. Adapted with permission from Ref. 88.

The slower recovery time constants for the dome and relaxed clusters were additionally confirmed by an analysis of the temporal evolution of the entire diffraction pattern, i.e., a so-called lifetime map. Each pixel of the temporal series of diffraction patterns is fitted by an exponential recovery function like that used in Fig. 12. The recovery time constant is displayed in Fig. 13(a) using color coding. Blue indicates diffracted intensity with fast recovery time constant up to 100 ps, red—recovery times about 250 ps, while green indicates slow time constant of 400–500 ps. Black areas indicate background intensity where the signal-to-noise ratio was insufficient to provide a proper fit. The blue to red asymmetry of all spots—i.e., blue is on the left, red in the middle, and green is on the right wing of all spots—supports the spot profile analysis we have performed before. This is shown more clearly in the inset of Fig. 13(a) showing the (117) spot.

The time constant of all pixels of the diffraction pattern from Fig. 13(a) is sorted in the histogram shown in Fig. 13(b). Three clear maxima can be identified at 40 ps, 160 ps, and 390 ps, which agree well with the recovery time constants for hut, dome, and relaxed clusters, which were independently determined from the spot profile analysis presented in Fig. 12.

### C. Debye–Waller effect at large momentum transfer: Electron–phonon coupling for Bi(111)

Bismuth is one of the prototypical model systems for studies of laser-induced energy transfer from an excited electron system to the lattice system in the time domain. Bi is a semimetal with the bottom of the conduction band slightly lower in energy than the top of the valence band. The almost vanishing density of states at the Fermi energy results in a low number of free carriers of 10^17^–10^19^ cm^−3^. This makes Bi very sensitive to optical excitations as changes in the electron occupation affects the potential energy surface and trigger atomic motion through displacive excitation. Bismuth exhibits a Peierls distortion, which breaks the translational symmetry along the (111) direction with every second Bi atom at a position slightly displaced from the center along the body diagonal of the unit cell. This equilibrium structure, in particular the distance of the two atoms of the basis, can easily be perturbed by electronic excitation,^111,112^ resulting in an oscillation of the Bi atoms along the body diagonal, i.e., the coherent symmetric A_1g_ optical phonon mode of the crystal.^113–118^

Depending on the degree of fs-laser optical irradiation, vastly different time constants for the excitation process of the Bi lattice were observed. Strong excitation with fluences of more than 6 mJ/cm^2^ generates so many electron–hole pairs that this causes a rapid change of the potential energy surface resulting in non-thermal melting. For fluences of 18 mJ/cm^2^, the electronic acceleration of the atomic motion occurs as fast as 190 fs, resulting in ultrafast melting, destruction of the Bi film, and a coherent A_1g_ phonon mode is not observed.^119,120^ For fluences lower than 6 mJ/cm^2^, the lattice response is reversible, the coherent A_1g_ optical phonon mode is excited,^114,116^ and bond softening occurs which results in an inverse Peierls transition.^115,120–123^ Subsequently, the lattice is heated on slower time scales of 2–4 ps (Refs. 116, 119, 124–126) through energy transfer from the electron system to the lattice by electron phonon coupling and anharmonic coupling of the A_1g_ mode to acoustic phonons.^127^ The vibrational excitation of the surface atoms is even slower: thermal motion of the Bi surface atoms sets in on a timescale of 12 ps and has been attributed to the weak coupling between bulk and surface phonons.^125^

Due to its high atomic mass and weak bonds, Bi exhibits a low-temperature bulk Debye temperature of only Θ_D_ = 112 K (Ref. 73) and thus a large vibrational amplitude upon the thermal motion. These large displacements make Bi an ideal model system to study lattice dynamics upon impulsive optical excitation by means of diffraction techniques. Here, we analyze the lattice excitation of the Bi(111) surface through tr-RHEED.

In earlier studies, the Debye–Waller effect was employed to follow the onset of atomic motion through the transient intensity changes of the diffraction patterns.^10,17,26,79^ Electron diffraction allows for a large momentum transfer due to the possible large scattering angles, which result in large intensity changes. Employing all detected diffraction spots of the RHEED pattern for the analysis provides a huge variation of the momentum transfer Δ**k** in diffraction, i.e., a wide range of parallel Δk_‖_ and vertical Δk_⊥_ momentum transfers are available all at once. Such analysis is presented here.

We followed the excitation of the surface lattice through the Debye–Waller effect *I/I*_0_ = exp −⟨(**ΔuΔk**)^2^⟩. For small intensity drop Δ*I*(*t*) = 1 − *I*(*t*)/*I*_0_ < 0.2, the intensity evolution *I*(*t*)/*I*_0_ can linearly be converted with an error of less than 6% in the time constant to a transient change of vibrational amplitude **Δu**(*t*) applying the linear expansion of the exponential function. This linear expansion, however, becomes inapplicable for intensity drops Δ*I*(*t*) > 0.2, which easily occurs for systems with a low Debye temperature, strong excitation, or diffraction at large momentum transfer Δ*k*. In such cases, the intensity *I*(*t*) decays with a time constant, which becomes significantly shorter with increasing intensity drop Δ*I*(*t*): a behavior that can easily be misinterpreted as fluence or temperature-dependent electron phonon coupling.

Here, we used RHEED spots on three different Laue circles, i.e., with different Δ*k*_‖_ and Δ*k*_⊥_, and various laser pump fluences for the excitation of the Bi(111) film in order to analyze the lattice dynamics of the Bi(111) surface. The non-linearity of the exponential function causes the decrease in the time constant τ_int_ for the decay of RHEED spot intensity from 11 to 5 ps with increasing laser fluence Φ and increasing momentum transfer Δ**k**. Irrespective of this large variation of τ_int_, we obtain a time constant of 12 ps for the heating of the Bismuth surface, which is independent of the level of excitation.

The 8-nm-thick epitaxial Bi(111) film was grown on a clean Si(111)–(7 × 7) reconstructed substrate.^6,128^ This sample is excited by 800 nm laser pulses at pump powers up to 1200 mW, corresponding to an incidence fluence of Φ = 2 mJ/cm^2^. A tilted-pulse-front scheme was used to compensate the velocity mismatch between electron and laser pulse. Here, we used electrons of 26 keV with a de Broglie wavelength of λ = 7.6 pm or momentum *k*_0_ = 2π/λ = 82.6 Å^−1^. They were diffracted at the sample under grazing incidence of 3.4°, i.e., resulting in a vertical momentum transfer of Δ*k*_⊥_ = 9.3 Å^−1^ for the specular (00) spot.

**FIG. 14.**
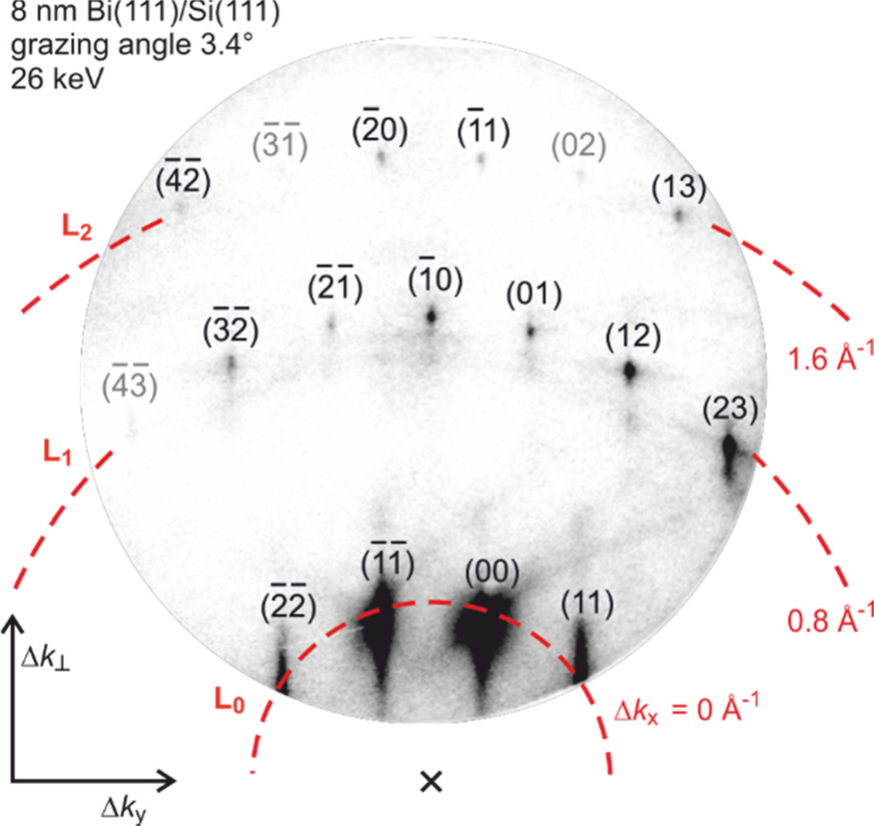
Diffraction pattern of a 8-nm-thick Bi(111) film on Si(111) recorded at an electron energy of 26 keV, a grazing angle of incidence of 3.4°, and a sample temperature of *T*_0_ = 90 K. The vertical Δ*k*_⊥_ and parallel Δ*k*_y_ momentum transfers of the diffracted electrons are indicated. All spots were identified using their Miller indices. The momentum transfer in the x direction along the incident electron beam (see Fig. 4) depends on the order of Laue circle (dashed lines indexed by L_0_, L_1_, L_2_). Adapted with permission from Ref. 58.

Figure 14 shows the diffraction pattern of the Bi(111)-film grown on Si(111) at a sample temperature of 90 K. The momentum transfer **Δk** is determined for all diffraction spots from diffraction geometry and reciprocal lattice constants. The diffraction pattern is shown in units of Δ*k*_⊥_ (left axis) and Δ*k*_y_ (bottom axis). Δ*k*_x_ increases with the order of Laue circles (dashed lines). Values for Δ*k*_⊥_ cover the range from 7 to 22 Å^−1^. The momentum transfer |Δ*k*_‖_| parallel to the surface is below 8 Å^−1^ for all observed spots. Since Δ*k*_⊥_ ≫ |Δ*k*_‖_|, our experiment is mainly sensitive to a change of the vibrational amplitude perpendicular to the surface.

**FIG. 15.**
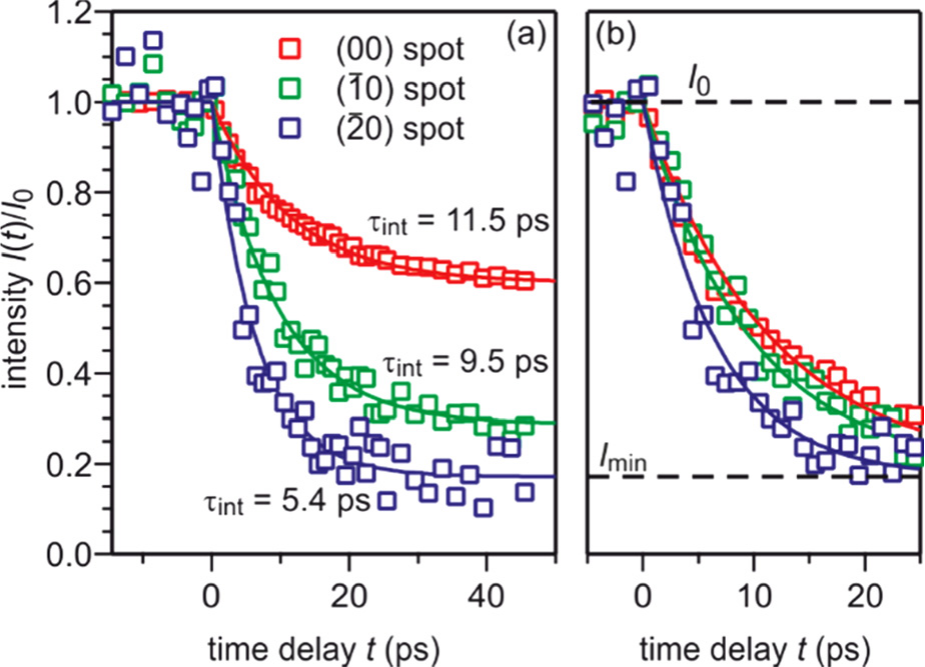
(a) The intensity *I*(*t*)/*I_t_*_0_ as function of the time delay is shown for three diffraction spots on different Laue circles (red: zeroth, green: first, blue: second) is shown. The intensity drop Δ*I*_max_ increases with momentum transfer from 40% to more than 80%, while the time constant decreases from 11.5 to 5.4 ps. (b) The intensity was normalized to the intensity drop to illustrate the difference in the time constants *τ*_int_. The incident pump laser fluence is Φ ≃ 1.4 mJ/cm^2^. Adapted with permission from Ref. 58.

In Fig. 15(a), the intensity evolution upon excitation with an incident pump laser fluence of Φ ≃ 1.4 mJ/cm^2^ is shown for diffraction spots on the three Laue circles: the (00) spot, the ( $\bar{1}$0) spot, and the ( $\bar{2}$0) spot. All diffraction spots exhibit an intensity drop that can be described by an exponential function. This intensity drop is caused by the Debye–Waller effect. The intensity decay *I*(*t*)/*I_t_*_0_ of the three diffraction spots in Fig. 15(a) scales with the squared momentum transfer that increases from 86.5 Å^−2^ for the (00) spot to 472 Å^−2^ for the ( $\bar{2}$0) spot. The time constant obtained from the exponential fit decreases from 11.5 ps for the (00) spot to 5.4 ps for the ( $\bar{2}0$) spot. To clearly illustrate this difference of the time constants, the normalized intensity drop Δ*I*(*t*) is plotted in Fig. 15(b).

In earlier works (see Sec. III A), the transient intensity drop was directly converted into a temperature curve employing a stationary calibration measurement.^51,79,80^ Here, we analyze the transient spot intensity without such conversion. For simplicity, we apply the Debye model in the high-temperature regime (*T* ≥ Θ_D,surf_) and assume an isotropic MSD ⟨*u*^2^⟩ proportional to the temperature as given in Eq. (7). We also assume an exponential increase in MSD, i.e., an exponential rise of temperature *T*(*t*) to a maximum temperature *T*_0_ + Δ*T*_max_, with a time constant τ_*heat*_,

$$T(t)=T_{0}+\Delta T_{\text{max}}\cdot \Theta (t)\;(1\;-\;\text{exp}\;(-t/\tau_{\text{heat}})).$$ (11)

The intensity is as follows:

$$I(t)/I_{0}=\;\text{exp}\;[-\alpha \Delta T_{\text{max}}\Theta (t)\;(1\;–\;\text{exp}\;(-t/\tau_{\text{heat}}))]$$ (12)

with α = 3ℏ^2^Δ*k*^2^/*mk_B_*Θ^2^_D,surf_. For small values of αΔ*T*_max_, we can safely use a linear approximation of the exponential because the higher-order terms in the expansion are negligible small

$$I(t)/I_{0}≃1-\alpha \Delta T_{\text{max}}\Theta (t)\;(1\;–\;\text{exp}\;(-t/\tau_{\text{heat}})).$$ (13)

With this approximation, the maximum intensity drop is Δ*I*_max_ = αΔ*T*_max_ and the time constant *τ*_int_—as experimentally determined from the transient intensity decay—is almost the same as *τ*_heat_ from the temperature curve. The question arises up to what arguments αΔ*T*_max_ we can use this linear approximation?

**FIG. 16.**
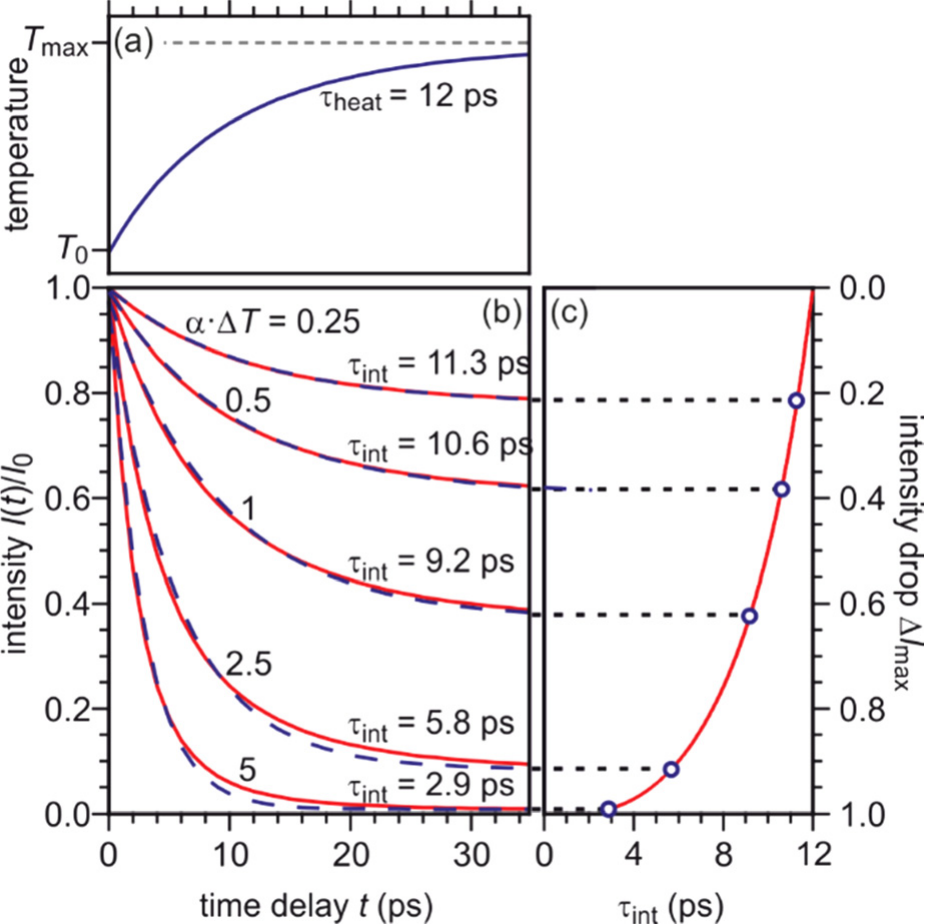
The intensity for an exponential temperature rise by Δ*T*_max_ with time constant τ_heat_ = 12 ps was modeled and is plotted as function of the time delay with different values of αΔ*T*_max_ (solid lines) on the left side. The curves are fitted by an exponential decay function (dashed lines). On the right side, the time constant obtained from the fit is plotted over the intensity drop Δ*I*_max_. With increasing values for αΔ*T*_max_, the intensity drop becomes larger and the fitted time constants decreases dramatically from 12 ps for αΔ*T*_max_ ≈ 0 to 2.9 ps for αΔ*T*_max_ = 5. Adapted with permission from Ref. 58.

We modeled the intensity to obtain the time constant τ_int_ in dependence of the intensity drop Δ*I*_max_. An exponential temperature rise with a time constant of τ_heat_ = 12 ps [see Fig. 16(a) and observed in Ref. 69] is converted into the corresponding intensity *I*(*t*) using Eq. (12). *I*(*t*) is plotted in Fig. 16(b) as function of the time delay for five different values of αΔ*T*_max_ (solid lines) and fitted with an exponential decay function (dashed lines). For small values αΔ*T*_max_ = 0.2, the calculated intensity *I*(*t*) exhibits almost the same behavior like *T*(*t*) and is well described by the fit function. The intensity drop Δ*I*_max_ is ≤ 18%, and the time constant obtained from the exponential fit (dashed line) τ_int_ = 11.3 ps deviates only by 6% from τ_heat_. With increasing values for αΔ*T*_max_, however, the time constant obtained from the exponential fit τ_int_ (dashed lines in Fig. 16) decreases. In the right panel of Fig. 16, the fitted time constant τ_int_ is plotted as function of the intensity drop Δ*I*_max_. For Δ*I*_max_ approaching unity, i.e., drop to intensity to almost zero, the time constant τ_int_ decreases to 3 ps and less. We therefore have to expect strongly varying experimental time constants τ_int_ depending on the degree of excitation (Δ*T*_max_) or momentum transfer (α). The varying time constants of 5.4–11.5 ps obtained for the different orders of Laue circles shown in Fig. 15 are thus explained by the correlation of Δ*I*_max_ and τ_int_ as shown in Fig. 16(c). The correct time constant of the temperature rise τ_int_ can only be found by extrapolation to Δ*I*_max_ = 0. Therefore, under our diffraction conditions at large momentum transfer Δ*k* and large intensity drop Δ*I*_max_, the time constants τ_int_ can be much shorter than τ_heat_. In the following, we perform a thorough Debye–Waller analysis in order to prove that the pre-conditions for such analysis are still valid. From the change of spot intensity, we obtain information about the change of the MSD

$$-ln(I(T)/I_{0})=1/3\Delta k^{2}(\langle u^{2}(T)\rangle -\langle {u_{0}}^{2}\rangle ).$$ (14)

**FIG. 17.**
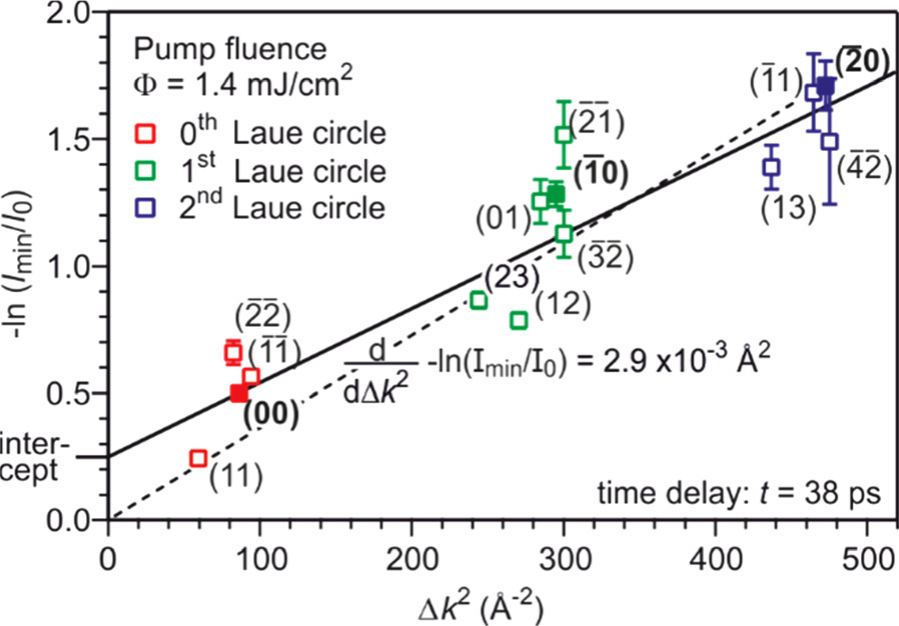
The negative logarithm of the minimum intensity *I*(*T*_max_)/*I*_0_ is plotted as function of Δ*k*^2^ for all diffraction spots at a time delay of *t* = 38 ps. Data from the different Laue circles are plotted in different colors. If applying kinematic scattering theory, a linear fit through the origin is expected (dashed line). The solid line gives a better fit to the data and the intercept is explained by multiple scattering effects. Adapted with permission from Ref. 58.

From kinematic diffraction theory,^68,69^ we expect a linear dependence of the negative logarithm of the intensity −ln(*I*(*T*)/*I*_0_) as function of Δ*k*^2^ with a y axis intercept equal to zero as evident from Eq. (14). The slope −d(ln(*I*(*T*))/*I*_0_)/*d*(Δ*k*^2^) is equal to one third of the change of the MSD Δ⟨*u*^2^⟩ = ⟨*u*^2^(*T*_max_)⟩ − ⟨*u*_0_^2^⟩ or, if the surface Debye temperature is known (here Θ_D,surf_ = 81 K), proportional to the temperature rise Δ*T*_max_, respectively. Figure 17 depicts −ln(*I*_min_/*I*_0_) for all diffraction spots plotted as function of the squared momentum transfer Δ*k*^2^. The value *I*_min_ is the minimum intensity obtained from the fit for the maximum transient temperature. The expected behavior for kinematic diffraction theory and isotropic vibrational motion is plotted as dashed line. The data are, however, better described by a linear fit with a y axis intercept >0. Such positive intercept was also observed in transmission electron diffraction experiments^129–131^ and is caused by multiple scattering effects. The offset observed in transmission electron diffraction was found to be proportional to the temperature change as well and is explained by dynamical two beam diffraction theory.

Though the data in Fig. 17 scatter around the linear slope, we did not find any systematic deviations as function of parallel Δ*k*_ǁ_ or vertical Δ*k*_⊥_ momentum transfer. This justifies the pre-assumption of an isotropic thermal motion. Thus, the present data do not provide insight into any potential non-equipartition in parallel or vertical vibrational amplitude. Finally, we obtain a change of the MSD at *t* = 38 ps that is Δ⟨*u*^2^⟩ = 8.8 × 10^−3^ Å^2^.

**FIG. 18.**
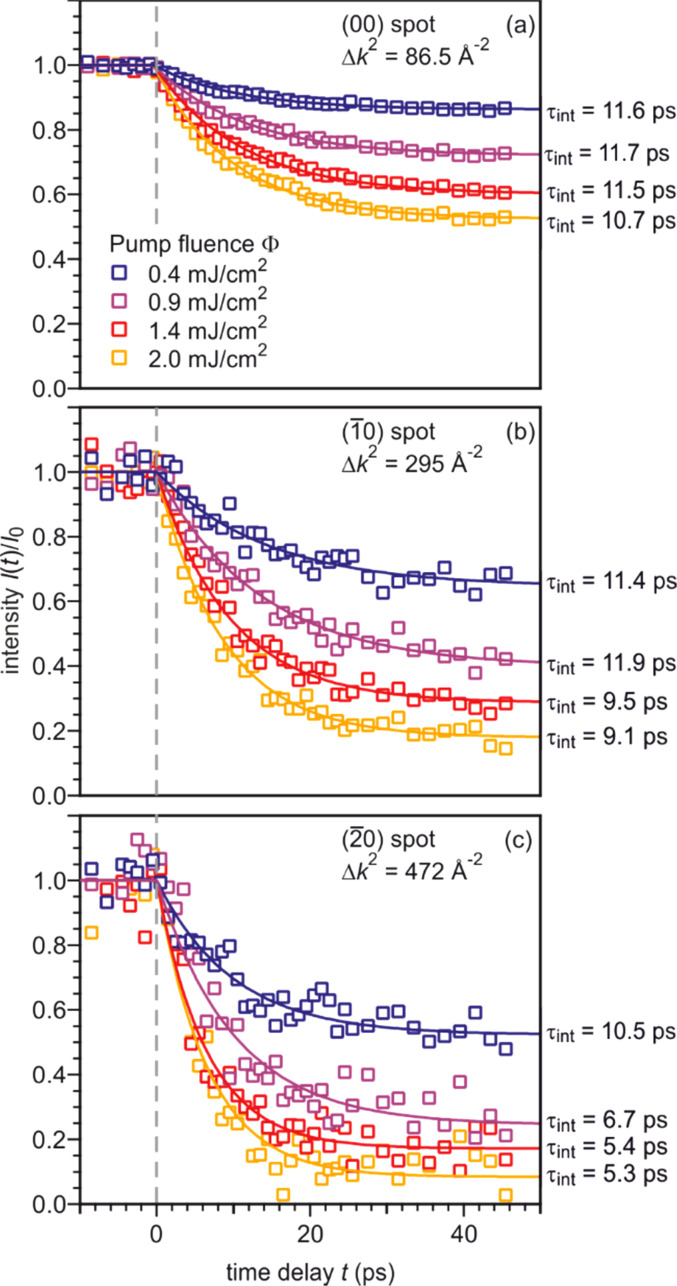
Transient intensity drops of the (00) ( $\bar{1}$0), and ( $\bar{2}$0) spot shown in (a)–(c) for increasing incident pump fluence, respectively. The time constant for an exponential fit to the data decreases with increasing momentum transfer Δ*k* and increasing excitation density. Adapted with permission from Ref. 58.

The intensity drop Δ*I*_max_ depends on the absorbed energy that was changed by varying the pump fluence. In Fig. 18, the intensity as function of the time delay is plotted for three diffraction spots (same as in Fig. 15) and four different pump fluences Φ between 0.4 and 2 mJ/cm^2^. The intensity drops Δ*I*_max_ becomes larger with increasing pump fluence for all diffraction spots.

For each pump fluence, a Debye–Waller analysis like in Fig. 17 was performed. The change of the MSD Δ⟨*u*^2^⟩ rises linear with the pump fluence.^58^ From this, we conclude that the absorbed energy is proportional to the pump fluence and the vibrational motion of the atoms is still in the harmonic regime of the potential. For the maximum laser pump fluence of Φ = 2 mJ/cm^2^, the MSD increases by Δ⟨*u*⟩ = 11.9 × 10^−3^ Å^2^. This corresponds to an asymptotic temperature rise of Δ*T*_max_ = 72 K.

**FIG. 19.**
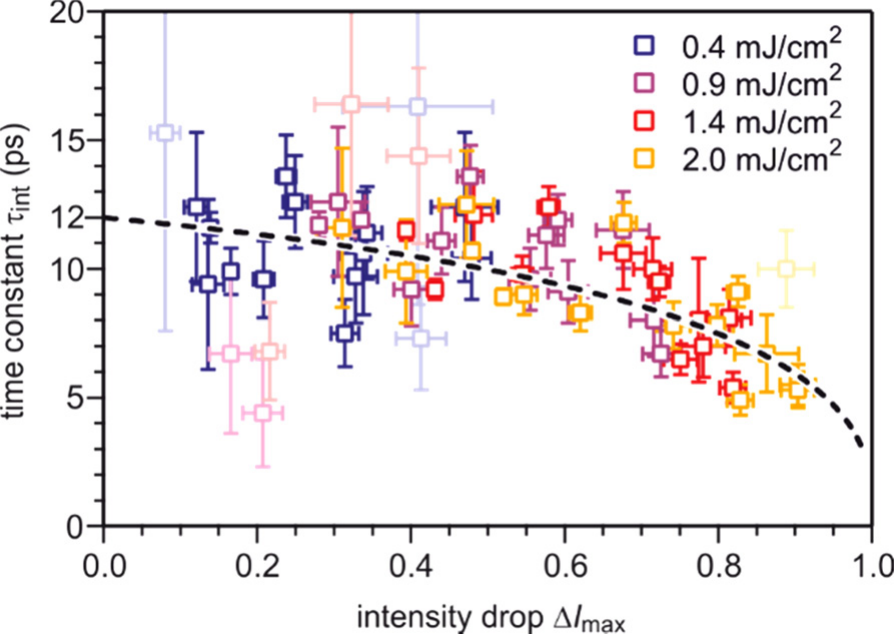
The measured time constant *τ*_int_ is plotted vs the intensity drop Δ*I* for all diffraction spots and all pump fluences. The light symbols represent weak diffraction spots with strong noise and large error bars. The dashed line is the expected behavior for a temperature rise Δ*T* with a time constant τ_heat_ = 12 ps. Adapted with permission from Ref. 58.

Increasing pump fluence and analysis of spots with larger momentum transfer Δ*k* result in strongly enhanced intensity drops Δ*I*_max_ and thus much shorter time constants τ_int_. The modeling shown in Fig. 16 explains this correlation well. Figure 19 summarizes all the experimental results and compares them with the expected behavior of τ_int_(Δ*I*) shown in Fig. 16 (dashed line). For each diffraction spot, the time constant determined from the fit is plotted over the intensity drop for all four pump fluences. For the determination of the time constant τ_heat_ of the temperature rise, we modeled τ_int_(Δ*I*_max_) curves for different values of τ_heat_ and found a minimum standard deviation for τ_heat_ = (12.0 ± 0.4) ps. No evidence for a dependence of excitation time constant τ_heat_ on the excitation level is found in the regime of weak excitation with incident pump fluences Φ ≤ 2 mJ/cm^2^.

We thus observe a time constant for heating of the surface atoms, which is more than four times larger than values reported for the bulk under conditions comparable to our incident laser fluences.^125^ Thus, the surface is not following the excitation of the bulk. Instead, the thermal excitation of the surface atoms occurs delayed on a timescale of τ_heat_ = 12 ps, which we attribute to a reduced electron phonon coupling at the surface. The Bi(111) surface exhibits a pronounced electronic surface state.^132–137^ This surface state is easily populated upon fs IR irradiation.^138^ The population of excited electrons in this surface state exhibits a lifetime comparable to the thermalization time constant observed in our experiment [see Fig. 5(a) of Ref. 138]. We conclude that slow energy transfer from the electronic surface state to the surface atoms is the dominant mechanism for the thermal excitation of the surface.

### D. Damping of vibrational excitations of monolayer adsorbate systems

Through excitation of localized vibrational modes in 2D adsorbate layers, it is possible to feed energy into a solid-state system at very high spatial selectivity. Transfer and dissipation of the deposited vibrational energy are topics of general interest, both from a fundamental and applied viewpoint, e.g., for controlling heat transfer through interfaces^139^ or chemical reactions^140^ at surfaces. Usually, the lifetime of vibrational modes is studied by means of infrared,^141^ sum frequency,^142–144^ or Raman spectroscopy.^145^ While each of these techniques has its specific advantages, the conservation laws and selection rules limit each technique to specific modes and regions of reciprocal space. Moreover, for heavy adsorbates, the vibrational frequencies are in the far infrared and thus difficult to access experimentally. In addition, diffraction methods have the advantage of being able to access spatial information as well as low-frequency vibrations since the vibrational amplitude *u* is proportional to 1/ω. Thus, topics like mode coupling in the adsorbate layer and to the substrate, which ultimately are responsible for the relaxation of the vibrational excitation can experimentally be accessed.

These processes have been studied for the vibrational dynamics of an ordered atomic layer of Pb atoms adsorbed on Si(111). Due to the large atomic mass of Pb and its low bulk modulus, very soft vibrational modes are expected. We will show that these modes couple to the acoustic phonons of the Si substrate only in a small sector of the two-dimensional Brillouin zone. Therefore, the acoustic phonons possess an unusually long lifetime of several nanoseconds and thus lend themselves as ideal objects for the study of relaxation by intermode coupling.

We employ the vectoral expression of the Debye–Waller effect

$$I(k)=\;\text{exp-}\langle {\left(\mathrm{ku}\right)}^{2}\rangle =\;\text{exp-}(\langle {\left(k_{\left|\right|}u_{\left|\right|}\right)}^{2}\rangle +\langle {\left(k_{⊥}u_{⊥}\right)}^{2}\rangle ),$$ (15)

where the scalar product of the vibrational displacement amplitude **u** and the momentum transfer **k** determines the sensitivity to a specific eigenmode. Because the (00) spot has no parallel momentum transfer, i.e., **k**_ǁ_ = 0, it is insensitive for any parallel component **u**_ǁ_ of the vibrational modes and only sensitive to the vertical component **u**_⊥_. Higher-order spots possess a parallel component of the momentum transfer **k**_ǁ_ ≠ 0 and are, therefore, also sensitive to the parallel component **u**_ǁ_ of the vibrational modes.

Here, we disentangle contributions from high-frequency in-plane **u**_ǁ_ from low-frequency out-of-plane **u**_⊥_ polarized vibrational modes, which exhibit clearly different dynamics in the time domain. Such approach allowed us to follow the conversion of the initial electronic excitation via strong electron-lattice coupling, the conversion of vibrational modes, and the dissipation of vibrational energy into the substrate.

The Pb layer with a coverage of 4/3 monolayer is prepared in the ( $\sqrt{3}$ × $\sqrt{3}$) phase by deposition on a clean Si(111)–(7 × 7) substrate at 90 K and subsequent annealing up to 700 K.^146–148^ Laser pulses with an incident fluence of 4.0 mJ*/*cm^2^ at a wavelength of 800 nm (1.55 eV) are used as pump pulses. Optical excitations of the Si substrate with its direct bandgap of 3.4 eV are still negligible at that fluence. The tr-RHEED experiments were performed with 7 keV electrons. The velocity mismatch reduces the temporal resolution to only 40 ps.^149^

**FIG. 20.**
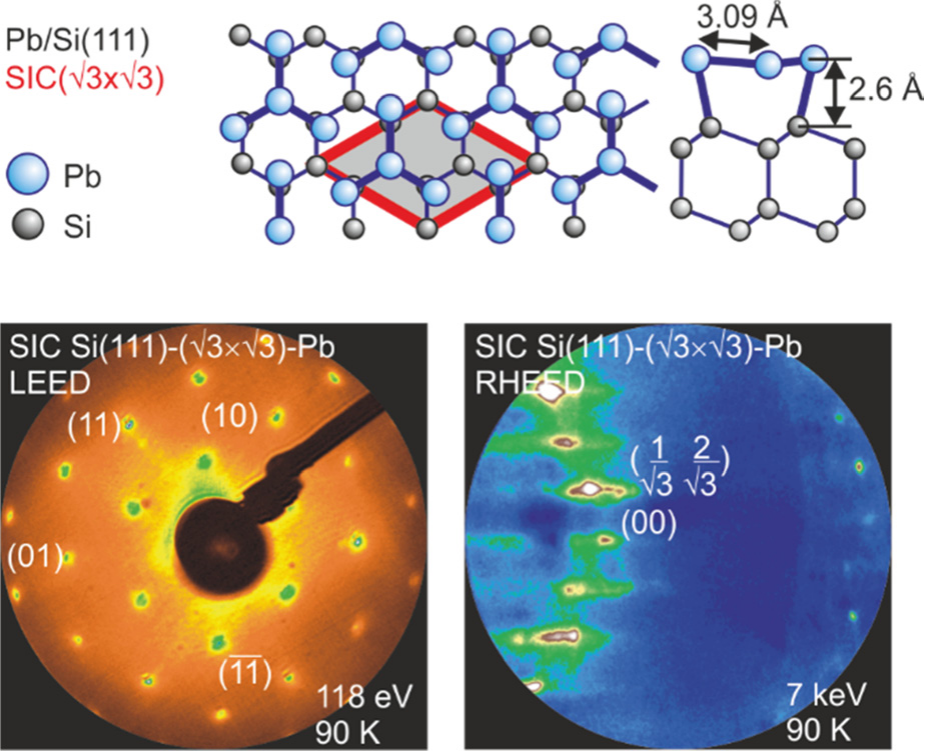
(a) Schematics of the atomic geometry of the SIC Si(111)–( $\sqrt{3}$ × $\sqrt{3}$)-Pb reconstruction. (b**)** LEED and (c) RHEED pattern of Si(111)–( $\sqrt{3}$ × $\sqrt{3}$)-Pb reconstruction on Si(111). The RHEED pattern shows integer-order and fractional-order spots located on the zero-order Laue circle. Adapted with permission from Ref. 47.

The relaxed geometric structure of the Pb ( $\sqrt{3}$ × $\sqrt{3}$) phase on Si(111) is presented in Fig. 20(a). The Pb layer exhibits an adsorption height of 2.6 Å.^149^ Each ( $\sqrt{3}$ × $\sqrt{3}$) unit cell possesses four Pb atoms, i.e., a saturation coverage of 4/3 of a Si(111) monolayer (ML = 7.8 × 10^14^ atoms/cm^2^). Three Pb atoms are bonded in a T1 site to the dangling bonds of the Si(111) surface, the remaining Pb atom in a T4 site bonds solely to the three other Pb atoms and is located in their center as sketched in Fig. 20(a). The two-dimensional electronic states induced by the metallic Pb layer are partially occupied and electrons from the filled Pb bands or from the Si substrate can be excited into the unoccupied Pb bands using infrared photons.^149^ As the Si bandgap prevents their diffusion into the substrate, the electrons will be deexcited by electron–electron and electron–phonon scattering within the Pb monolayer, thus exciting Pb phonons. LEED and RHEED patterns are shown in Figs. 20(b) and 20(c), respectively.

**FIG. 21.**
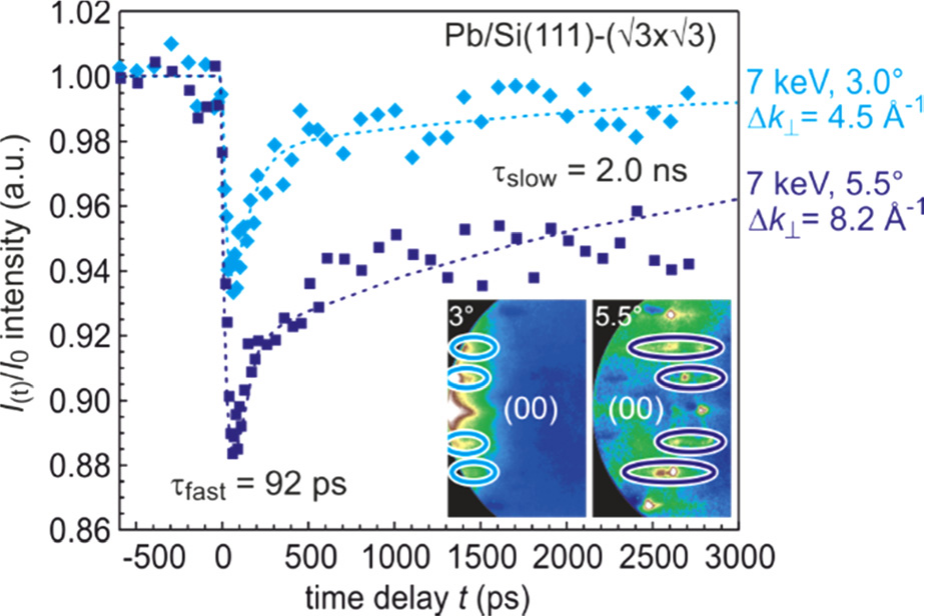
The transient intensity drop of the ( $\sqrt{3}$ × $\sqrt{3}$) spots upon excitation by a femtosecond-laser pulse exhibits a bi-exponential recovery with a fast (τ_fast_ ≅ 100 ps) and slow component (τ_slow_ ≅ 2 ns). Data for grazing angles of 3° and 5.5° are shown. Inset: RHEED patterns of the Pb-induced ( $\sqrt{3}$ × $\sqrt{3}$) phase on Si(111) at 90 K. Those ( $\sqrt{3}$ × $\sqrt{3}$) spots used for the analysis are encircled. Adapted with permission from Ref. 149.

In tr-RHEED, the transient intensities of individual diffraction spots were analyzed by integration of a small elongated region of interest around each spot, which are marked in Fig. 21 and normalized by the number of pixels. The (00) spot was excluded from the analysis as it may be affected by contributions from the Si substrate and still existing Pb islands. Multiple runs were performed at varying grazing angles between 3° and 5.5°, thus allowing for a variation of perpendicular momentum transfer from Δ*k*_⊥_ = 4.5 to 8.2 Å^−1^. Figure 21 shows for grazing angles of incidence of 3° and 5.5° transients of the intensity of a ( $\sqrt{3}$ × $\sqrt{3}$) diffraction spot, displaying a sharp drop, followed by a slow recovery that extends over nanoseconds.

The bi-exponential recovery of the ground state is described by a fast Δ*I*_fast_ and slow Δ*I*_slow_ component of the intensity drop

$$I(t)/I_{0}=1-Q(t)\;[\Delta I_{\text{fast}}\;\text{exp}(-t/\tau_{\text{fast}})+\Delta I_{\text{slow}}\;\text{exp}(-t/\tau_{\text{slow}})],$$ (16)

where Θ(*t*) is the Heaviside function that defines temporal overlap at *t* = 0. The fast contribution exhibits a decay constant τ_fast_ ≤ 100 ps, while the much slower contribution relaxes with a decay constant τ_slow_ ≥ 2000 ps. Fitting the experimental data with Eq. (16) yields very similar time constants for the recovery of intensity for all diffraction angles, and hence, by averaging all angles, we find the values of τ_fast_ = 92 ± 8 ps and τ_slow_ = 2007 ± 267 ps. This fit is shown in Fig. 21 as dashed lines together with the data for incident angles between 3.0° and 5.5°. We note that the time constants τ_fast_ and τ_slow_ are markedly different, i.e., the corresponding relaxation processes must be significantly different.

**FIG. 22.**
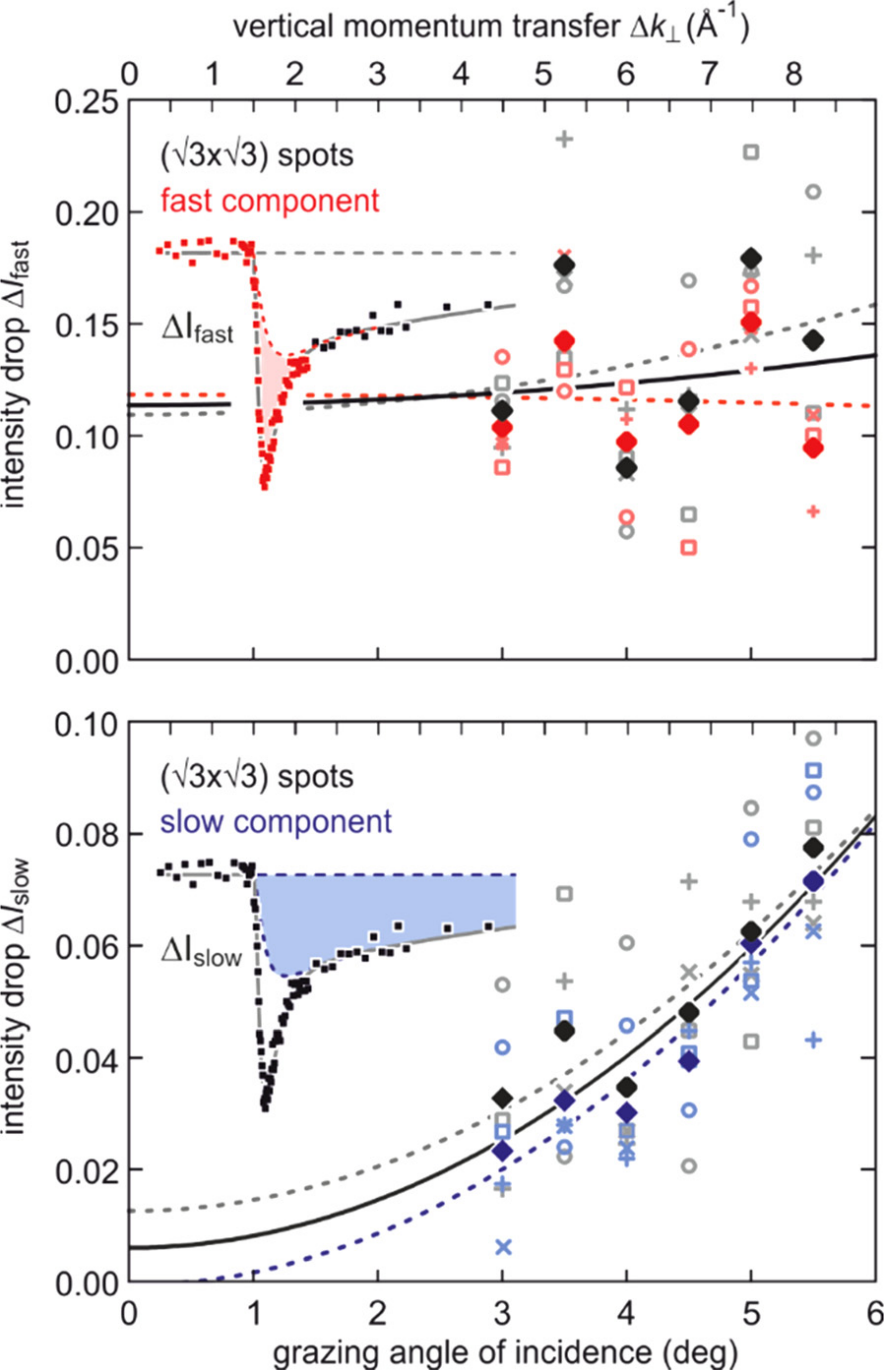
Coefficients for the fast Δ*I*_fast_(θ) and slow Δ*I*_slow_(θ) component of the Debye–Waller effect as a function of the grazing angle θ of the electron beam. Open symbols display intensity drops of individual ( $\sqrt{3}$ × $\sqrt{3}$) diffraction spots. Solid symbols display averaged data. Solid lines are a parabolic fit to the data. Dotted lines give error margins of fit. Adapted with permission from Ref. 149.

The corresponding intensity coefficients Δ*I*_fast_(θ) and Δ*I*_slow_(θ) are shown in Fig. 22 as a function of the grazing angle θ. We notice that Δ*I*_fast_(θ) is independent of θ, whereas Δ*I*_slow_(θ) increases with θ. We cannot exclude that the data points are affected by multiple scattering effects, i.e., maxima in the rocking curves and thus providing an additional modulation of the data, which might show up as increased scattering. The overall trend of constant and rising intensity as function of vertical momentum transfer for the fast and slow component is not affected, respectively. According to Eq. (15), the intensity coefficient is proportional to the mean square of the Pb displacement vectors projected onto the momentum transfer Δ**k**. Because Δ**k** is predominantly perpendicular to the surface in our experiment, we are mostly sensitive to perpendicular adsorbate vibrations. From Fig. 22(a), we are led to the conclusion that the Pb displacements, contributing to Δ*I*_fast_, are almost entirely within the surface plane, and hence insensitive to the amount of perpendicular momentum transferred in the diffraction. We determine a dominant change of the parallel mean squared displacement of Δ⟨*u*_ǁ_^2^⟩ = 0.025 Å^2^.

The long-lived vibrational excitations in the Pb layer that contribute to Δ*I*_slow_ therefore has to exhibit a strong perpendicular component in order to explain the observed angular dependence with Δ*I*_slow_ ∼ Δ*k*_⊥_^2^. The parabolic dependence on vertical momentum transfer Δ*k*_⊥_ is expected from the expansion of Eq. (15) for small intensity decreases Δ*I* < 0.2. We determine a change of the vertical mean squared displacement of Δ⟨*u*_⊥_^2^⟩ = 0.0016 Å^2^. From the almost vanishing intercept of *ΔI*_slow_(*k*_⊥_) with the ordinate at *k*_⊥_ = 0, we conclude a small parallel component for the slow contribution only. A parallel component of the long-lived vibrations, however, cannot be completely ruled out, as the experimental diffraction geometry with *k*_⊥_ > *k*_ǁ_ results in a higher sensitivity to perpendicular as to parallel displacements of the Pb atoms.

Because the data in Fig. 22 suggest that initially no out-of-plane modes are excited, the decay of the optical Pb modes into substrate modes must be accompanied by a conversion of a small fraction of the excitation into low-energy out-of-plane modes. From the value of Δ*I*_slow_ together with a measurement of the static Debye–Waller effect, we estimate the rise of temperature in the Pb system of Δ*T* = 20 K after thermalization of the phonon system for times beyond 100 ps.

In summary, we find that upon irradiation with the fs-laser pulse, initially only modes with dominant parallel vibrational amplitudes *u*_ǁ_ ≫ *u*_⊥_ are excited. These modes convert on a timescale of τ_fast_ ≤ 100 ps to modes that exhibit a dominant vertical vibrational amplitude *u*_⊥_ ≫ *u*_ǁ_.

**FIG. 23.**
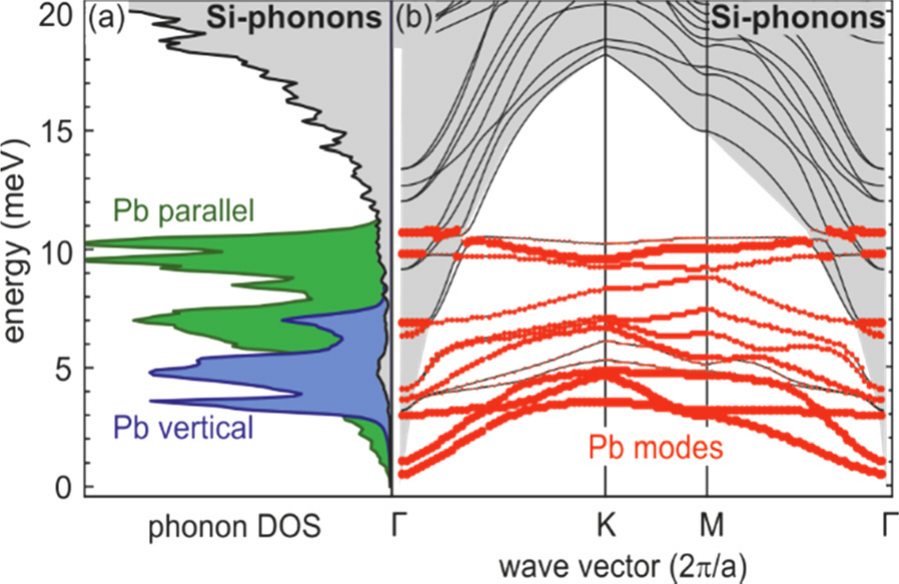
(a) Mode-specific phonon DOS for the Pb layer and for the Si substrate. Parallel and vertical modes are shown in green and blue, respectively. (b) Phonon dispersion for the Pb layer (red dots) and the Si substrate in gray. Adapted with permission from Ref. 149.

The subsequent excitation of the fast and slow modes and their two significantly different de-excitation time constants can be explained through electron–phonon coupling in the Pb layer and phonon–phonon coupling from Pb layer to the Si substrate. Figure 23(a) depicts the phonon dispersion of the Pb layer in solid red lines as function of wave vector with a maximum energy of the phonons at 10 meV. Most of these modes may be denoted as optical modes with a finite energy of 4–10 meV at the Γ-point. There are only three acoustic phonon branches with *E*(Γ) = 0 at the zone center and *E*(Κ) = 5 meV at the zone boundary.

Only a small fraction of the phonon phase space can be excited through the excited electron system since the conservation of energy and momentum must be obeyed. Typical values for the dispersion in the electron system of the states in the Pb layer are Δ*E*_el_/Δ*q* ∼ 0.1–2 eV/Å^−1^ (see Fig. 3 in Ref. 149), while typical values necessary for the generation of acoustic phonons are smaller than *E*_ph_/*q* < 0.005 eV/Å^−1^ as shown in Fig. 23(a). As a consequence, only optical phonons with almost vanishing wave vector and finite energy fulfill the requirement of conservation of energy and momentum with q < *E*_ph_/0.1 eV/Å^−1^ ∼ 0.05 Å^−1^ during excitation. The acoustic phonons already carry a too large momentum at finite energy and can thus not be excited.

The mode selected density of phonon states (DOS) is shown in Fig. 23(b) and reveals that the initially excited high-frequency optical modes exhibit displacements only parallel to the surface plane without vertical component. These modes are observed as fast component in diffraction. The modes with vertical displacements can be associated with the zone boundary optical and acoustic branches,^149^ which initially are not excited. These modes finally become populated through mode conversion.

In the framework of the diffuse mismatch model, the cooling toward the Si substrate is determined by the overlap of the Pb and Si phonon DOS as shown in Fig. 23(b). As the parallel modes **u**_ǁ_ exhibit large overlap with the Si modes, they decay quickly on a 100 ps timescale into the Si substrate. In contrast, the overlap of the vertical modes **u**_⊥_ with the Si modes is much smaller, and consequently, the decay of these modes needs more than 2 ns, which has also been corroborated by molecular dynamics simulations.^149^

### E. Driven structural transition at a surface: Melting of a CDW in atomic wire system Si(111)-In (8 × 2)↔(4 × 1)

**FIG. 24.**
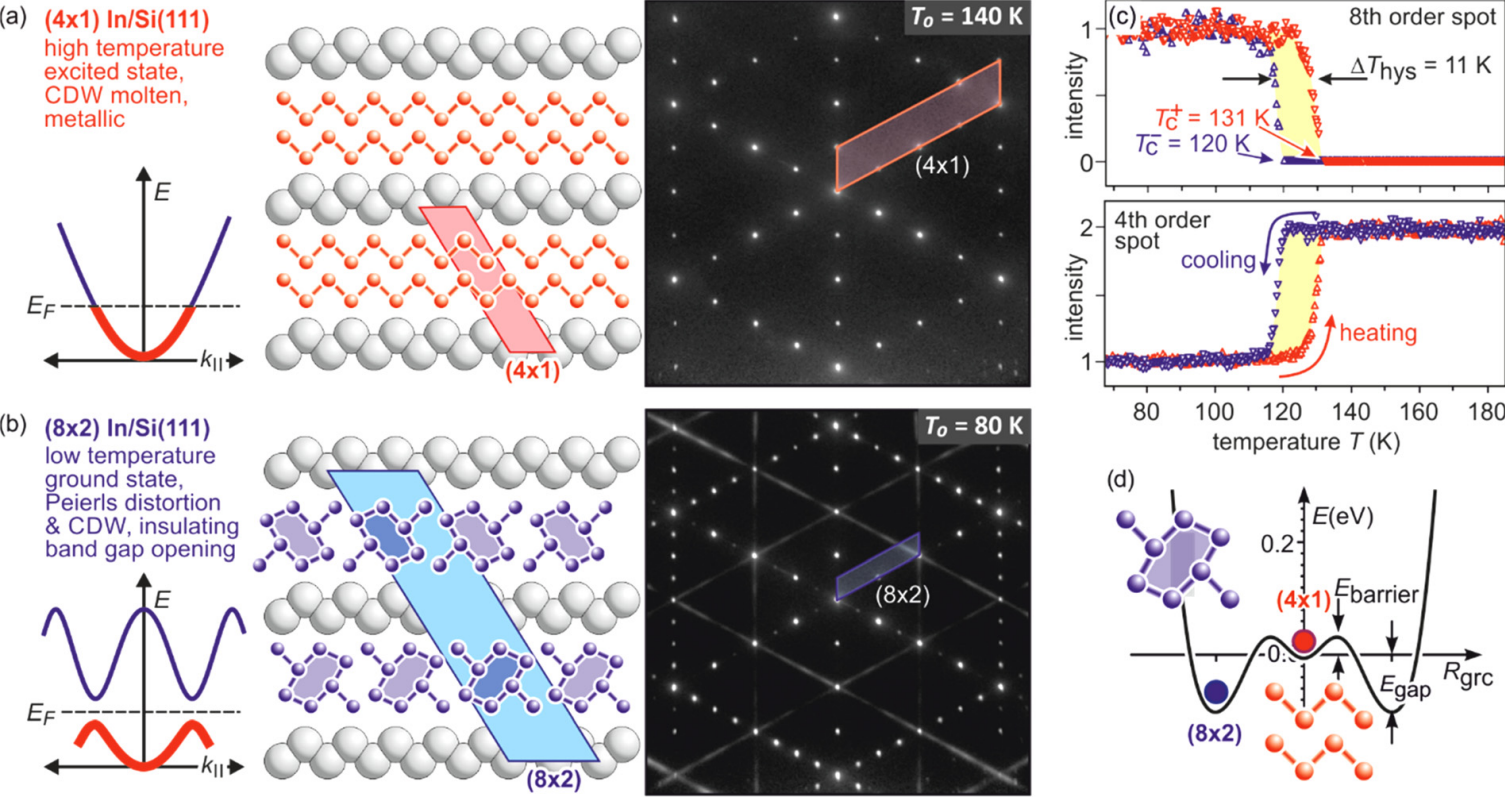
(a) The metallic high-temperature (4 × 1) state is composed of In atoms arranged in double zigzag chains. The LEED pattern depicts the (4 × 1) reconstruction in three rotational domains. (b) The insulating (8 × 2) ground state exhibits a Peierls distortion with the formation of a CDW and opening of a bandgap. The In atoms are rearranged in distorted hexagons. The (8 × 2) LEED pattern clearly shows the periodicity doubling along and perpendicular to the wires. The eightfold spots and twofold streaks are indicative for the ground state structure. (c) RHEED intensity of the (8 × 2) spot (upper panel) and the (4 × 1) spot (lower panel) as function of temperature. Upon heating, the intensity of the (8 × 2) spot drops to the background at *T*_c_. Cooling with the same rate leads to the transition back into the (8 × 2) reconstruction. The intensity of the (4 × 1) spot rises upon heating at *T*_c_, reflecting the change of atom position in the unit cell. The temperature cycling of both spots exhibits a hysteresis of 11 K. (d) Potential energy surface obtained through density functional calculations as function of a generalized reaction coordinate *R*_grc_ describing the transition from the (4 × 1) and the (8 × 2) phase. The blue and red dots indicate the (8 × 2) ground state and the metastable (4 × 1) state, respectively. Adapted with permission from Ref. 168.

**FIG. 25.**
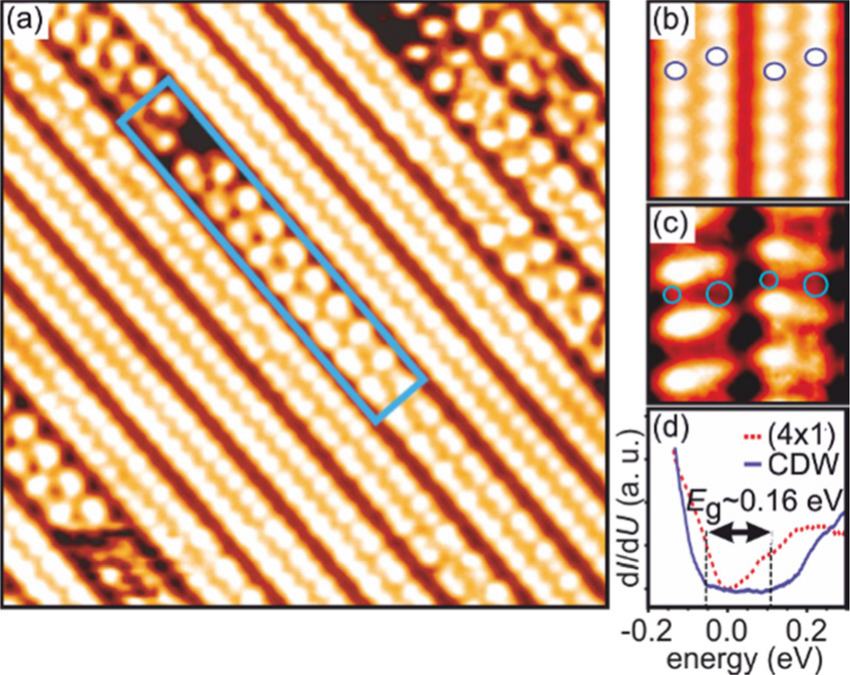
(a) STM micrograph under constant current conditions taken at 100 K. Both (8 × 2) and (4 × 1) reconstructed indium wires can be seen. (b) The metallic (4 × 1) wires are composed of In atoms arranged in two parallel zigzag chains. (c) Instead, in the insulating (8 × 2) wires the chains are broken up and distorted hexagons of In atoms form. (d) STS spectra for the (8 × 2) and (4 × 1) reconstructed wires. While the (4 × 1) exhibit metallic behavior (dashed red line), the (8 × 2) clearly shows opening of a bandgap of *E*_gap_ = 0.16 eV (solid blue line). Data courtesy of H.W. Yeom and adapted with permission from Ref. 155.

Due to its unique and peculiar properties, the indium atomic wire system is ideally suited for the study of structural and electronic dynamics at surfaces. This system exhibits an inherent Peierls instability manifesting itself in a first-order phase transition between an insulating (8 × 2) ground state and metallic (4 × 1) high-temperature state. This structural transition can non-thermally be driven through an optical excitation and subsequently is trapped for nanoseconds in a supercooled metastable state. The indium atomic wire system is prepared by self-assembly under ultrahigh vacuum conditions.^150–156^ *In situ* deposition of a monolayer (1 ML is equivalent to 7.83 × 10^14^ cm^2^ atoms) of indium on Si(111) substrates at a sample temperature of 700–750 K creates the (4 × 1) In/Si(111) reconstruction. The metallic high-temperature phase of this atomic wire system is composed of two parallel zigzag chains of indium atoms with a (4 × 1) unit cell^157,158^ as is sketched in Fig. 24(a). The corresponding low-energy electron diffraction (LEED) pattern is shown in the right panel of Fig. 24(a) with its threefold symmetry arising from three rotational domains at the hexagonal (111) surface. Upon cooling the system undergoes a reversible phase transition from the metallic high-temperature state to the insulating ground state^152,159,160^ at *T*_c_ = 130 K (Refs. 155, 161, 162), which is accompanied by the opening of a bandgap of *E*_gap_ = 0.2 eV (Refs. 155, 163) and the formation of a charge density wave (CDW). In the ground state, the zigzag chains of indium atoms are broken and they rearrange into distorted hexagons,^157,158^ as sketched in Fig. 24(b). Upon this phase transition, the maximum change of geometric position of the In atoms in the surface unit cell is less than 0.1 Å only.^158^ This Peierls-like transition is characterized by symmetry breaking in both directions, which is facilitated through soft shear- and rotational-phonon modes, with frequencies of *v*_shear_ = 0.54 THz and *v*_rot_ = 0.81 THz, respectively.^157,164–167^ The surface periodicity doubles along and normal to the wires and the size of the unit cell increases to (8 × 2). This change becomes obvious in the LEED pattern in the right panel of Fig. 24(b) through the appearance of additional spots at eightfold position between the fourfold spots. The appearance of twofold streaks emerge from the broken correlation of the twofold periodicity in neighbored wires. The anisotropic nature of the indium atomic wire system becomes immediately apparent in scanning tunneling microscopy (STM). Figure 25(a) displays a filled-state STM image from Ref. 155 from the indium wire surface at *T* = 135 K, i.e., at *T*_c_. Extended and parallel wires both with (8 × 2) and (4 × 1) reconstruction are present. Employing scanning tunneling spectroscopy both at the (8 × 2) and (4 × 1) structure—as shown in Figs. 25(b) and 25(c)—reveals the opening of a bandgap of *E*_gap_ = 0.16 eV for the low-temperature (8 × 2) structure—see Fig. 25(d)—and is indicative for the formation of a charge density wave and the metal to insulator transition.^155^

The equilibrium phase transition has been followed during quasi-stationary rise of temperature from 70 to 180 K where the sharp drop of intensity of the eightfold spots to zero (see upper panel of Fig. 24(c) is indicative for the transition from the (8 × 2) ground state to the (4 × 1) high-temperature state. At the same time, the intensity of the fourfold spots sharply rises by a factor of two and thus indicating the structural transition as shown in the lower panel of Fig. 24(c). During slow temperature cycling, a hysteresis of the high-temperature (4 × 1) and low-temperature (8 × 2) states is observed upon heating and cooling as shown in Fig. 24(c). The width of the hysteresis is independent of the cooling/heating rate d*T*/d*t.*^161^ Such behavior is evidence of a first-order phase transition, i.e., a non-continuous transition with both states separated by a small energy barrier.

#### 1. Photo-induced “phase transition”

**FIG. 26.**
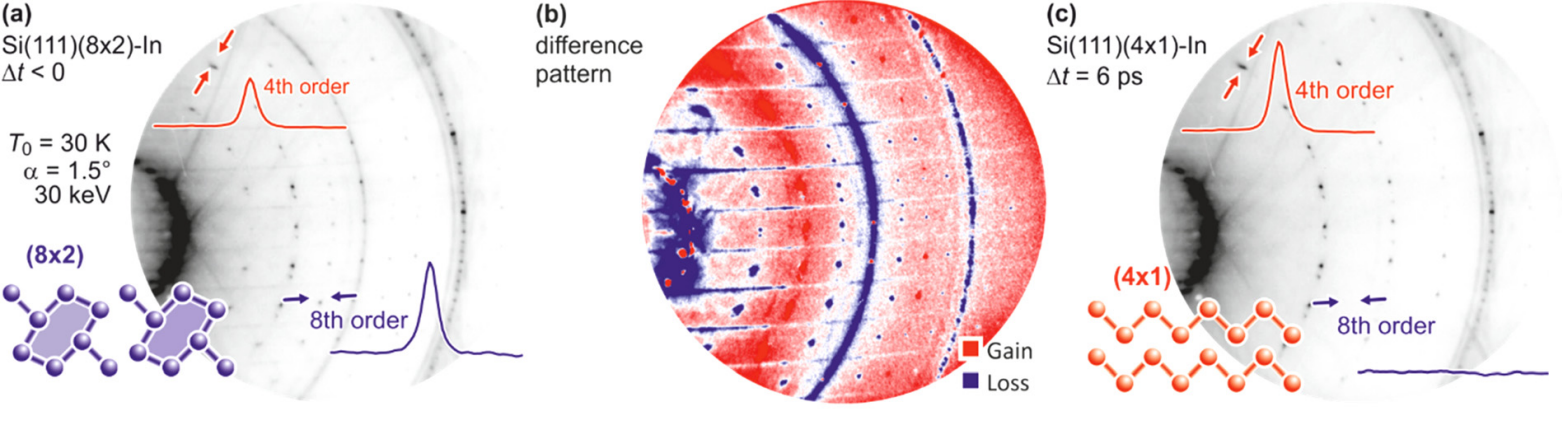
RHEED patterns for clarity shown in inverted intensity representation (bright spots are shown in the dark, background in bright) at 30 K prior and after optical excitation through a fs-laser pulse. (a) Pattern exhibiting (8 × 2) ground state. Spot profiles of a fourfold and an eightfold spot are shown in red and blue, respectively. (c) The pattern 6 ps after excitation has changed to (4 × 1). All (8 × 2) spots and twofold streaks are disappeared, as evident from the changes in spot profile, indicating the structural transition. (b) The difference pattern in false color representation exhibits systematic changes: all (4 × 1) spots gain intensity (red) while the (8 × 2) and twofold streaks disappeared (blue). Adapted with permission from Ref. 168.

This structural transition can be triggered by impulsive optical excitation through intense laser pulses (1–10 mJ/cm^2^ on a femtosecond timescale of 50–120 fs). The sudden and massive optical excitation of the electron system transiently changes the potential energy surface for the atoms position in the lattice. This provokes accelerating forces on the atoms ultimately causing the structural transition. This photo-induced structural transition is demonstrated in Fig. 26 where panel (a) depicts the RHEED pattern of the (8 × 2) ground state prior to optical excitation at negative pump–probe delays Δ*t* < 0 at a temperature *T*_0_ = 30 K, i.e., well below *T*_c_ = 130 K. The pattern taken at Δ*t* = 6 ps, i.e., after optical excitation through a fs-laser pulse with a fluence of Φ = 6.7 mJ/cm^2^, is shown in panel (c) and exhibits clear differences. The transient changes of spot intensity become more obvious in the difference pattern in panel (b) depicting intensity gains (red) and losses (blue) in a false color representation. All eightfold spots and twofold streaks (indicative for the ground state) disappeared, while fourfold spots (indicative for the high-temperature state) gained intensity. The complete transition from the (8 × 2) ground state to the (4 × 1) excited state is also reflected by the clear changes in the two representative spot profiles shown for a (8 × 2) and (4 × 1) spot in blue and red, respectively.^169^

#### 2. Supercooled excited state

**FIG. 27.**
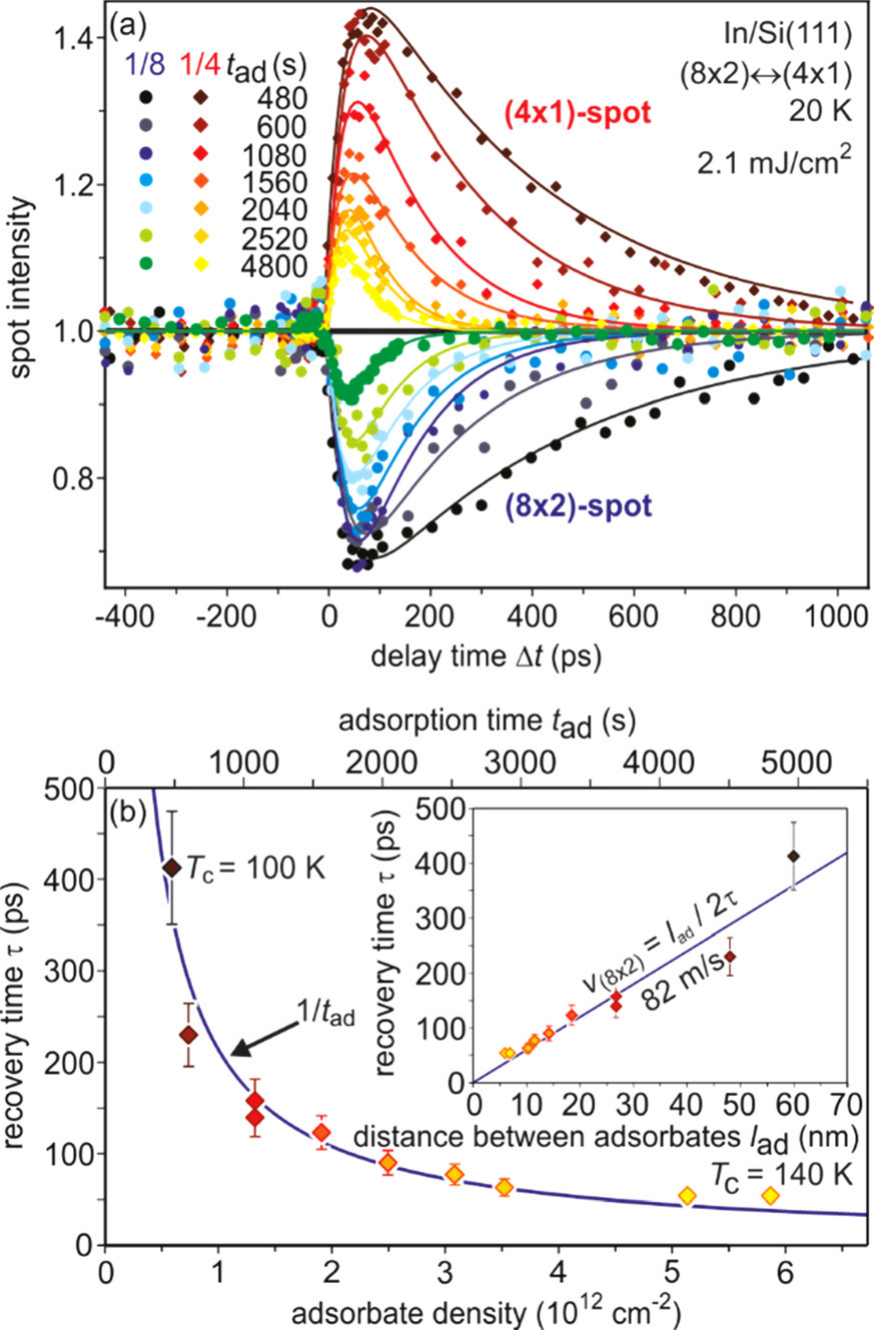
Recovery of the (8 × 2) ground state. (a) The recovery of the (8 × 2) ground state strongly depends on adsorption from the residual gas. With increasing adsorbate density, the recovery time constant τ changes from τ = 415 ps for the first experiment at *t*_ad_ = 480 s (dark red data points) to τ = 54 ps at *t*_ad_ = 4800 s (light yellow data points). (b) Time constant t for the recovery of the (8 × 2) reconstruction as a function of adsorbate density. The solid line describes a 1/*t*_ad_ behavior. From the slope in the inset, we derive a velocity of the propagating phase front of *v*_(8 × 2)_ = 82 m/s. Adapted with permission from Ref. 163.

Surprisingly, the excited (4 × 1) state is stable for ns and only slowly recovers the (8 × 2) ground state as shown in Fig. 29(f), where the intensity of a (4 × 1) spot is plotted for long pump–probe delays Δ*t*. As we show later, the indium surface layer cools via heat transport on a τ_cool_ = 30 ps timescale to the substrate temperature of *T*_0_ = 30 K. We thus can safely exclude a slow thermal recovery of the (8 × 2) ground state. This long-lived (4 × 1) state is explained through the nature of the equilibrium phase transition: in general, a first-order transition exhibits a barrier between the two states hindering the immediate recovery of the ground state. This picture is corroborated through density functional calculations of the potential energy surface (PES). Figure 24(d) depicts this PES as function of a generalized reaction coordinate *R*_grc_ obtained by superimposing the soft shear and rotary phonon eigenvectors that transform between the (4 × 1) and the (8 × 2) phase.^151,157^ We found the transition from the (4 × 1) phase back to the (8 × 2) phase to be hampered by an energy barrier of *E*_barrier_ = 40 meV (see Fig. 24). At temperatures below *T*_c,_ this barrier hinders the immediate recovery to the (8 × 2) ground state: A long-lived metastable and supercooled excited phase is stabilized and trapped in a state far from equilibrium for few nanoseconds.^163,169^ In analogy to a supercooled liquid, one might even expect the freezing, i.e., the transition back to the (8 × 2) ground state, to be facilitated by condensation nuclei, possibly in form of adsorbates. To verify this assumption experimentally, we manipulated the dynamics of the structural transition through controlled adsorption of molecules from the residual gas. The transient intensity evolution of the (8 × 2) (black to green dots) and (4 × 1) spots (red to yellow dots) is plotted in Fig. 27(a) for various adsorption times *t*_ad_. With increasing adsorbate coverage, we observed a strong decrease in the time constant, as depicted in Fig. 27(b).

**FIG. 28.**
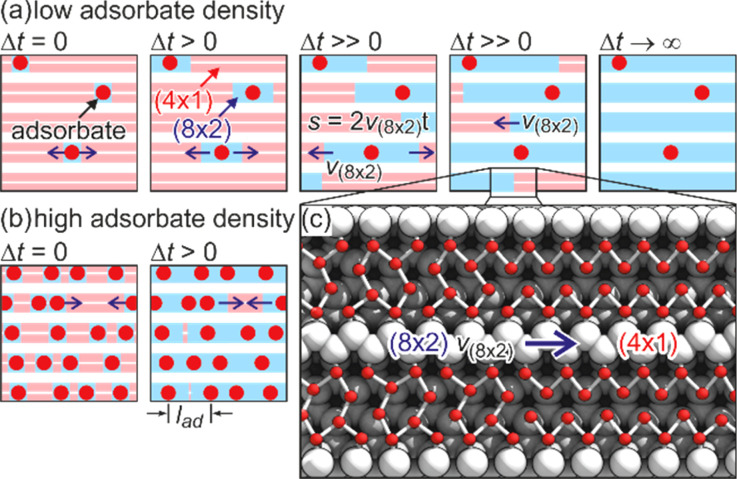
Propagation of the phase front of the (8 × 2) ground state. (a + b) Adsorbates with a mean separation *l*_ad_ act as seeds (red dots). *v*_(8 × 2)_ is the velocity of the propagating phase front. Low (a) and high (b) adsorbate densities are shown. (c) A snapshot from the *ab initio* molecular dynamics simulations depicts the transition from the metastable (4 × 1) phase to the (8 × 2) ground state. Adapted with permission from Ref. 163.

The shortest observed time constant was τ = 54 ps for an adsorption time of *t*_ad_ = 75 min. The solid line shows a fit to a 1/*t*_ad_ behavior. Obviously, the adsorption from the residual gas drastically shortens the recovery time of the (8 × 2) ground state by almost a factor of 10. Sticking to the analogy with a supercooled liquid, the insertion of seeds, i.e., condensation nuclei, initiates the freezing, which then propagates with constant velocity. Here, freezing means recovery of the (8 × 2) ground state. Because of the highly anisotropic nature of the indium-induced Si surface reconstruction, this phase front propagates only one-dimensionally along the direction of the indium chains. Therefore, the velocity of the phase front *v*_(8 × 2)_ within the one-dimensional In wire and the averaged distance *l*_ad_ between the condensation nuclei determine the time constant *τ* for the complete recovery of the (8 × 2) ground state: τ = *l*_ad_/(2 *v*_(8 × 2)_), as sketched in Fig. 28. In addition, assuming a linear relation between adsorbate coverage Θ_ad_ and the time *t*_ad_, the distance between the adsorbates in one row obeys *l*_ad_ ∝ *t*_ad_^−1^; consequently, it holds τ ∝ *t*_ad_^−1^. This is indeed the experimental finding shown in Fig. 27(b). An estimate for the distance *l*_ad_ between adsorbates in one individual row can be obtained from the shift of critical temperature *T*_c_ as a function of the adsorbate density Θ_ad_. We observed Δ*T* = +40 K after adsorption for *t*_ad_ = 75 min. According to Lee and Shibasaki, such a change in *T*_c_ is induced by an adsorbate density of Θ_ad_ = 6 × 10^12^ cm^−2^ as determined by scanning tunneling microscopy.^162,170^ The distance lad between the adsorbates, together with the measured time constant *τ*, is sufficient to determine the lower limit of the phase front velocity *v*_(8 × 2)_. The present experimental data result in a value of *v*_(8 × 2)_ = 82 m/s.^163^

The transition back to the (8 × 2) ground state may also be facilitated by condensation nuclei in form of the omnipresent steps on the Si(111) substrate.^171^ With the knowledge of the mean terrace width between two atomic steps ⟨Γ⟩ = 350 nm and the time constant of recovery to the ground state τ_rec_ = 3 ns, a speed of the 1D-recovery front of 112 m/s^2^ was determined experimentally,^171^ which agrees with the above determined value of *v*_(8 × 2)_ = 82 m/s.

#### 3. Melting of a CDW at the quantum limit

**FIG. 29.**
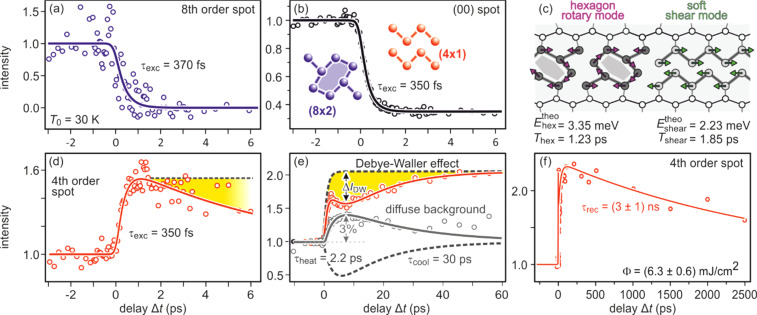
Time evolution of the diffraction intensities following the fs-photoexcitation as a function of pump–probe delay Δ*t*. Solid lines are (exponential) fits to the data. (a) The transient intensity of an (8 × 2) spot at a laser fluence of Φ = 6.7 mJ/cm^2^ vanishes at a rate of τ_exc_ = 370 fs to the background level. (b) Transient intensity of the (00) spot reflecting the structural transition from (8 × 2) to (4 × 1) state at a rate of τ_exc_ = 350 fs. (c) Characteristic hexagon rotary and soft shear phonon modes facilitating the transition. (d + e) Intensity of a fourth-order spot and the thermal diffuse background at a laser fluence of Φ = 6.7 mJ/cm^2^. The transient dip in the intensity of the fourth-order spot Δ*I*_DBW_ (yellow shaded area) at Δ*t* = 6 ps indicates surface heating by Δ*T* = 80 K, which coincides with the increase in background intensity. The fourth-order spot intensity is described (solid red curve) by the superposition of the two dashed lines, representing incoherent thermal motion (heating and subsequent cooling with time constants of 2.2 and 30 ps, respectively) and the structural transition with τ_exc_ = 350 fs. (f) Metastable state for long timescales. The supercooled (4 × 1) state recovers slowly on a 3 ns timescale. Adapted with permission from Ref. 168.

**FIG. 30.**
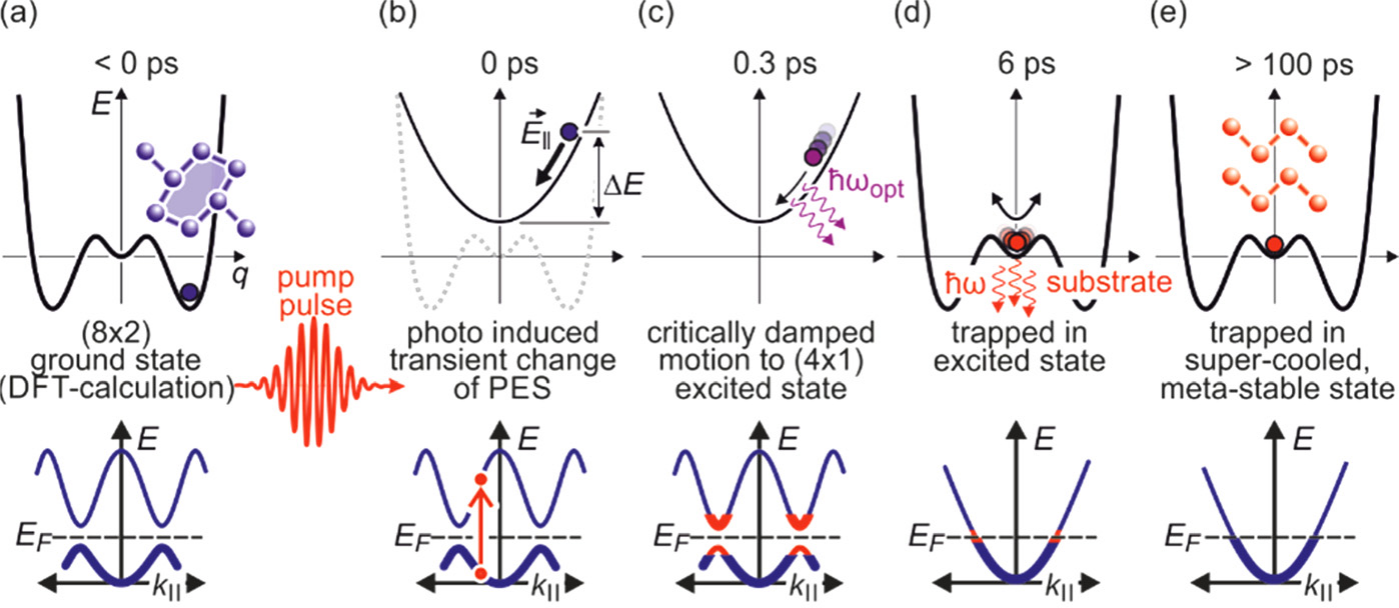
Sketch of transient changes of potential energy surface (upper row) and simplified band structure (lower row) as function of time delay Δ*t*. (a) ground state prior to excitation. (b) Photo excitation, generation of electron–hole pair, excitation of electron system, transient change of PES. (c) Accelerated displacive structural transition, critically damped motion due to effective energy dissipation to manifold of surface phonon modes. (d) System is trapped in excited high-temperature state, electron, and lattice system are thermalized. (e) Ground-state PES, system trapped in metastable, supercooled state, energy barrier hinders immediate recovery of ground state for nanoseconds. Adapted with permission from Ref. 168.

The initial dynamics of this optically driven structural transition was followed in the time domain through the transient intensity changes of RHEED spots as function of pump–probe delay Δ*t*. Figure 29(a) shows that the eighth-order diffraction intensity (indicative for the ground state) is quenched in less than 1 ps. Owing to the much higher signal-to-noise ratio as compared to the eighth-order spots, the dynamics of the more intense (00) spot was analyzed, which follows the same trend as the eighth-order spots. The (00) spot decreases with a time constant of τ_trans_ = 350 fs for a laser fluence of Φ = 6.7 mJ/cm^2^ as shown in Fig. 29(b), i.e., the structural transition is completed in only 700 fs. No oscillatory signatures of the optical phonons connected to the periodic lattice distortion are observed, in contrast to studies on other CDW materials.^172–174^ This structural transition from the initial insulating (8 × 2) state to the final metallic (4 × 1) state is driven by transient changes of the ground-state PES, which is sketched in Fig. 30(a). Photo excitation of the electron system leads to a depopulation of those states at the top of the surface state valence band, which are responsible for the energy gain through the Peierls distortion as sketched in Fig. 30(b). This results in a transient change of the energy landscape, as is sketched in Fig. 30(c) for Δ*t* = 0.3 ps. Inevitably, the system undergoes a strongly accelerated displacive structural transition to the minimum of the transient energy landscape. The transition from (8 × 2) state to the excited (4 × 1) state is completed after 0.7 ps. The temporal fine structure of one of the (4 × 1) diffraction spots as shown in Figs. 29(c) and 29(d) is determined by two opposing trends. First, the initial increase within less than 1 ps is due to the structure factor enhancement of the (4 × 1) phase reflecting the change of atomic position. Second, the subsequent decrease in intensity is explained by the Debye–Waller effect and results from the excitation of incoherent surface vibrations.^68,69^ This leads to a transient minimum at 6 ps, which is confirmed by the rise of the thermal diffuse background and its temporal evolution (gray circles). We find time constants of 2.2 ps and 30 ps for heating and cooling of the indium atoms, respectively. This situation is sketched in Figs. 30(d) and 30(e) for Δ*t* = 6 ps and Δ*t* > 100 ps. From the stationary Debye–Waller behavior of the high temperature (4 × 1) phase and its extrapolation to lower temperatures, we determined the maximum transient temperature *T*_max_ = *T*_0_ + Δ*T*_max_ = 30 + 80 K = 110 K at Δ*t* = 6 ps, which is below *T*_c_ = 130 K. We therefore conclude that the structural transition occurs with τ_exc_ = 350 fs, well before the initial excitation has thermalized at 6 ps, and thus is not thermally driven.

**FIG. 31.**
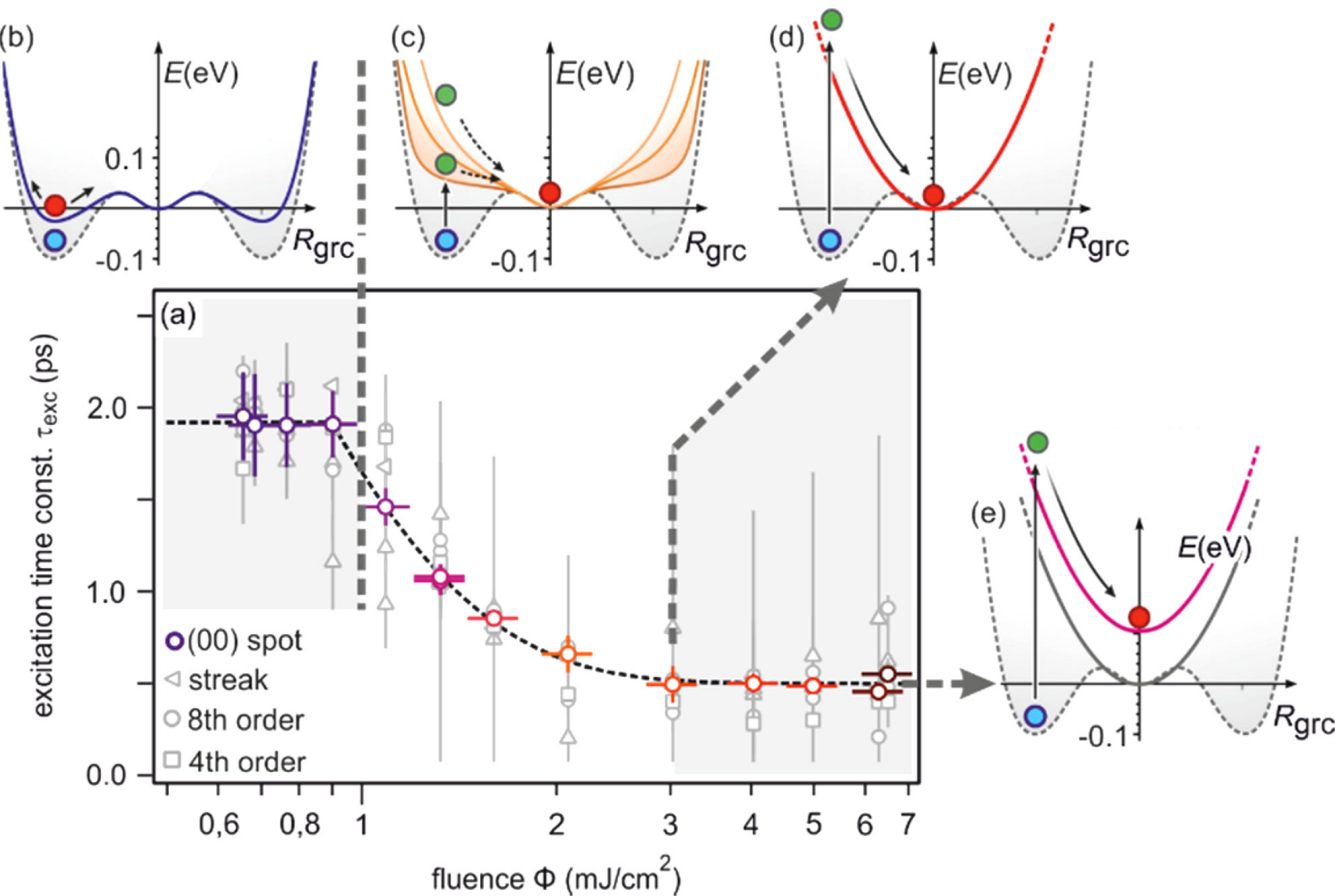
(a) Fluence dependence of the excitation time constant τ_exc_ of the driven structural transition. Below Φ = 0.9 mJ/cm^2^ the (8 × 2) state is not driven into the excited (4 × 1) state. The ground state exhibits excitation of the CDW as sketched in (b). For the intermediate regime 0.9 mJ/cm^2^ < Φ < 3 mJ/cm^2^, the accelerated displacive structural transition into the excited (4 × 1) state takes place. The slope of the transient PES increases, i.e., speeding up the transition as is sketched in (c). The transition speed saturates for Φ ≥ 3 mJ/cm^2^. The slope of the transient PES is maximum as sketched in (d) and (e). Adapted with permission from Ref. 168.

We observe a threshold fluence of 1 mJ/cm^2^ below which the system shows some transient response but does not make it into the excited (4 × 1) state. The excitation of the characteristic shear and rotational-phonon modes at a frequency of 0.82 and 0.54 THz has been indirectly observed in a pump–pump–probe experiment addressing coherent control of the structural transition.^45^ The resulting amplitude mode is sketched in Fig. 31(b).

**FIG. 32.**
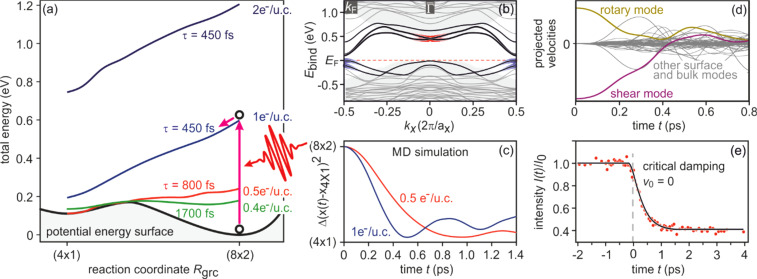
Potential energy surfaces, electronic surface states, molecular dynamics. (a) Calculated potential energy surfaces for the ground state (black) and various excited configurations along the (8 × 2) → (4 × 1) minimum-energy path, i.e., along the generalized reaction coordinate *R*_grc_. The open circles and purple and red arrows indicate excitation of the (8 × 2) phase. (b) Calculated electronic bands of the Si(111)(8 × 2)–In surface. Here, *k*_x_ and *a*_x_ are the reciprocal- and real-space lattice vectors in the wire direction; *E*_bind_ is the electron binding energy relative to the valence band maximum in silicon. The electron occupation of the blue and red shaded surface bands (black lines) is vital for the structural transition. Gray-shaded areas show projected silicon bulk bands. (c) Time evolution of the structural deviation from the (4 × 1) state, obtained from *ab initio* molecular dynamics simulations within the adiabatic approximation, for two excited configurations. Here, *x*(t) and *x*(4 × 1) denote the atomic coordinates of the In atoms, during the molecular dynamics calculation and for the high-temperature phase, respectively. (d) Transient atomic velocities projected onto vibrational eigenmodes. As evident, the rotary and shear modes rapidly transfer their energy to other modes. (e) The transient intensity *I*(*t*)/*I*_0_ of the (00) spot is well fitted by the behavior expected for critical damping (solid black line). Adapted with permission from Ref. 169.

For an incidence fluence larger than 1 mJ/cm^2^, the potential energy surface of the (8 × 2) ground state is transiently lifted above the barrier between the two states as sketched in Fig. 31(c). The surface system undergoes an accelerated transition to the excited (4 × 1) state in a displacive excitation scenario. Naturally, with increasing laser fluence, i.e., increasing excitation density, the slope of the PES becomes steeper resulting in a faster transition to the excited state, as predicted by theory. The transition time constant saturates for incident fluences of 3 mJ/cm^2^ or more as depicted in Fig. 31(d). This behavior nicely confirms the theoretical predictions where higher excitations densities only shift the PES without increasing its gradient as predicted by theory, see Fig. 32(a). The observed sharp transition from the initial (8 × 2) state to the final (4 × 1) state, without any sign of damped oscillatory behavior, is explained through fast mode conversion, dephasing and strong damping of the two characteristic rotary and shear phonon modes.^169,175^ Figure 32(d) shows that the initially excited rotary and shear modes rapidly transfer their energy to a manifold of modes at the surface of the Si substrate. The experimentally determined asymptotic value of τ_trans_ = 350 ± 10 fs is about 1/4 of the periods of the equilibrium rotational and shear modes, *T*_rot_ = 1.2 ps and *T*_shear_ = 1.8 ps,^157^ respectively. Consequently, the transition proceeds in the regime of critical damping and cannot be faster than in this limit. All of the surface indium atoms move in a spatially coherent manner: the system undergoes the structural transition in the quantum limit: “Quantum limit is the non-statistical regime of rates in which the nuclear motion is directed and deterministic on the shortest scales of length (0.1–1 nm) and time (10^−13^ to 10^−12^ s).”^26^ Our results demonstrate that structural transitions at surfaces can be driven as fast as those in bulk materials in the non-thermal regime.

The optically induced Si(111)(8 × 2)–In CDW melting relies on transient changes in the PES that arise from the population of very specific electronic states. These directly couple to the two characteristic rotational and shear vibrational lattice modes that drive the structural transition. This melting mechanism is similar to the structural bottleneck mechanism in layered bulk materials,^176^ which is surprising because the surface system differs from bulk CDWs in one important detail. Bulk CDWs are formed within layers or chains that only weakly couple to the environment, and so, the signatures of low-dimensional physics remain intact. In contrast, the surface CDW analyzed here is characterized by In–In and In–Si bonds that are stronger than In–In bulk bonds. Despite this strong interaction within the surface and between surface and substrate, the Peierls instability remains. The substrate serves as a skeleton that anchors the indium atoms, but with sufficient freedom to adopt different lateral positions. The strong coupling between substrate and adsorbate facilitates the sub-picosecond structural response by dephasing and damping the characteristic phonons after the structural transition. The structural transition of CDW melting at the Si(111)(8 × 2)–In surface therefore proceeds in a non-thermal regime in a limit of critical damping of the atomic motion. The CDW interaction with the surface enables the transition timescale to be controlled via the coupling strength of surface atoms to the environment and opens up possibilities for using femtosecond switching to control and steer energy and matter.

## IV. CONCLUSIONS

Ultrafast time-resolved RHEED is a versatile tool to study relaxation processes of excited surface systems on a picosecond to femtosecond timescale. In this review, we have demonstrated that possible applications range from heat transport in nanostructures via mode conversion in adsorbate layers to the non-equilibrium dynamics of driven “phase transitions.” Whenever structural dynamics is considered, ultrafast RHEED will provide new and often unforeseen insights into the non-equilibrium processes at surfaces.

Improved temporal resolution was achieved through the implementation of a tilted-pulse-front scheme to compensate the velocity mismatch between probing 30 keV electrons and pumping laser pulse. Ultimately, an unprecedented temporal response of the entire experiment of 330 fs full width at half maximum of the temporal instrumental response function was obtained.

## Data Availability

The data that support the findings of this study are available from the corresponding author upon reasonable request.
